# Stereoretention in the Bulk ROP of l-Lactide Guided by a Thermally Stable Organocatalyst

**DOI:** 10.1021/acs.macromol.1c01060

**Published:** 2021-06-16

**Authors:** Andere Basterretxea, Elena Gabirondo, Coralie Jehanno, Haijin Zhu, Olivier Coulembier, David Mecerreyes, Haritz Sardon

**Affiliations:** † POLYMAT, University of the Basque Country UPV/EHU, Paseo Manuel de Lardizabal 3, 20018, Donostia-San Sebastian, Spain; ‡ Institute for Frontier Materials, Deakin University Waurn Ponds Campus, Geelong, VIC 3220, Australia; § Center of Innovation and Research in Materials and Polymers (CIRMAP), Laboratory of Polymeric and Composite Materials, University of Mons, Place du Parc 23, 7000 Mons, Belgium; ∥ IKERBASQUE Basque Foundation for Science, 48009 Bilbao, Spain

## Abstract

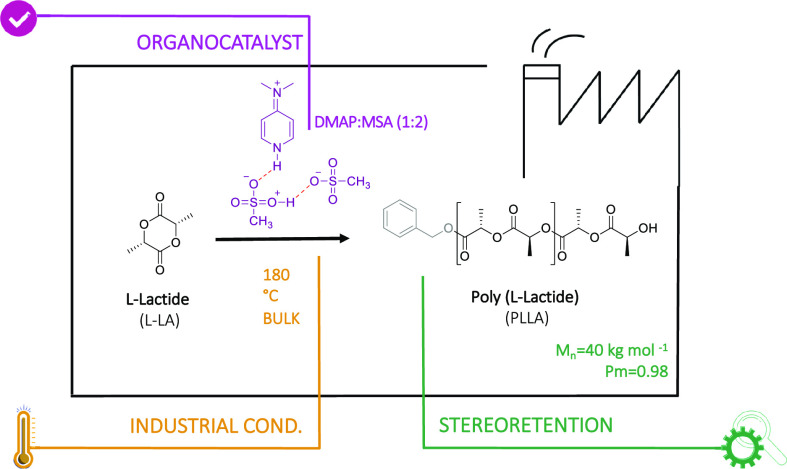

Polylactide (PLA) has emerged as one of the most promising bio-based alternatives to petroleum-based plastics, mainly because it can be produced from the fermentation of naturally occurring sugars and because it can be industrially compostable. In spite of these benefits, the industrial ring-opening polymerization (ROP) of l-lactide (L-LA) still requires the use of highly active and thermally stable metal-based catalysts, which have raised some environmental concerns. While the excellent balance between activity and functional group compatibility of organic acid catalysts makes them some of the most suitable catalysts for the metal-free ROP of L-LA, the majority of these acids are highly volatile and subject to decomposition at high temperature, which limits their use under industrially relevant conditions. In this work we exploit the use of a nonstoichiometric acid–base organocatalyst to promote the solvent-free and metal-free ROP of L-LA at elevated temperatures in the absence of epimerization and transesterification. To do so, a stable acidic complex was prepared by mixing 4-(dimethylamino)pyridine (DMAP) with 2 equiv of methanesulfonic acid (MSA). Both experimental and computational results indicate that DMAP:MSA (1:2) not only is highly thermally stable but also promotes the retention of stereoregularity during the polymerization of L-LA, leading to PLLA with a molar mass of up to 40 kg mol^–1^ and a chiral purity in excess of 98%. This result provides a new feature to exploit in organocatalyzed polymerization and in the design of new catalysts to facilitate the path to market.

## Introduction

To overcome the problems associated with our current linear plastic production model, the development of sustainable polymers is a central topic in which catalysis plays a major role. The development of efficient and versatile catalytic transformations will be necessary to convert our demand of nonrenewable materials into a circular economic model. This model encourages the development of polymers that are derived from renewable feedstocks and that exhibit closed-loop life cycles. Aliphatic polyesters and polycarbonates have been considered excellent candidates to meet these two criteria as they can be produced from biomass and have shown great potential to be circular-by-design.^1,2^ These polymers are obtained by ring-opening polymerization (ROP) of their corresponding cyclic monomers, and in all cases, the catalyst is the key parameter to accelerate the polymerization while maintaining control over the reaction.^3−5^ Although in the past 20 years a great variety of catalysts have been investigated for such transformations, most of these compounds operate at low temperatures and in the presence of solvent, two factors which have limited their industrial implementation.

One of the most studied sustainable polymers is polylactide (PLA) because it is both biodegradable and obtained from a renewable monomer. On top of this, it has been demonstrated that PLA is a suitable substitute for many commodity plastics synthesized from petroleum derivatives which makes it a good target for commercial applications.^6−8^ PLA is industrially produced from the ROP of lactide (LA), a transformation requiring high temperatures and the intervention of an efficient catalyst.^6,9^ Despite the continuous efforts to implement sustainable catalysis in its production, the current synthetic pathway requires metal catalysts, the most widespread example being tin octoate (SnOct_2_), employed because of its excellent thermal stability and high activity even at low catalyst loading. However, the subsequent removal of SnOct_2_ is arduous, which regularly compromises the applicability of PLA and generates environmental issues at the end of the useful life of the plastic when it is left to biodegrade.^10−13^ In addition, at the industrial level, concerns have been raised due to upcoming regulations limiting the use of tin-based catalysts.^14^

As an alternative, a substantial amount of work has been directed toward the use of organocatalysts for the synthesis of PLA.^15,16^ Unfortunately, such catalysts demonstrate poor thermal stability, which is particularly significant given the high temperatures typically required for the ROP of LA, and gives rise to some undesired side reactions such as epimerization or transesterification. One strategy to address the challenge of the catalyst’s thermal stability which is gaining attention is the use of organocatalysts based on hydrogen bond donor–acceptor adducts.^17−20^ These catalysts have recently demonstrated to be stable and active at elevated temperatures, in some cases at temperatures above 400 °C.^18^ On the basis of the pioneering work of Lin and Waymouth on deprotonated ureas as catalysts,^21^ Kiesewetter and co-workers have investigated these systems for the ROP of LA at moderate temperatures, up to 110 °C.^22^ They found that some monoureas and bisureas in combination with organic bases could operate at such temperatures while maintaining good control over the polymerization. More recently, Peruch et al. have explored different acids in combination with 4-(dimethylamino)pyridine (DMAP) in the presence of protic initiators at 100 °C.^23^ The mixture involving triflic acid and DMAP in excess displayed outstanding catalytic activity and was able to mediate the synthesis of PLLA with molecular weights up to 14 kg mol^–1^ in 1 h. However, the reaction control was reduced upon elevating the temperature. Similarly, by use of DMAP and saccharin, a naturally occurring acid, in stoichiometric quantities, the ROP of L-LA provided stereoregular PLLA of moderate molecular weights (*M*_n_ = 4 kg mol^–1^) but narrow dispersity at 90 °C.^24^

Besides possessing good thermal stability and remaining stable under harsh conditions, the employed catalyst must be able to limit the side reactions typically occurring in the ROP of LA such as epimerization, transesterification, and macrocyclization, which are known to diminish the thermal and mechanical properties of the resulting PLA, especially if L-LA is employed with the objective of obtaining stereoregular PLLA.^25,26^ This is particularly important when seeking for highly crystalline polyesters. In this respect, the excellent balance between activity and functional group compatibility of organic acid makes them some of the most suitable catalysts for the defect-free ROP of L-LA.^27−29^ However, the majority of these acids are highly volatile and subject to decomposition at high temperature, thus limiting their potential application in such polymerization processes.

In this work, we have designed a stable nonstoichiometric acid–base mixture with acid character for the ROP of L-LA at industrially relevant temperatures, i.e., up to 180 °C. It was established that DMAP, which has already shown potential for the ROP of LA, can form an acidic complex with two methanesulfonic acid (MSA) molecules. Thermal characterization indicates that DMAP:MSA (1:2) is significantly more stable than the stoichiometric mixture DMAP:MSA (1:1), which was corroborated by DFT calculations. Taking advantage of the acidic character of the catalyst, we investigated its potential for the high-temperature polymerization of L-LA. DMAP:MSA (1:2) performs above expectations by mediating the ROP of L-LA, resulting in PLLA with high molecular weights (up to 40 kg mol^–1^) and controlled stereoregularity (chiral purity up to 98%). DFT calculations help to elucidate the mechanistic aspects to justify the controlled stereoregularity in the presence of DMAP:MSA (1:2). Finally, we show that this catalyst not only is efficient for the polymerization of L-LA but also allows block copolymers to be prepared by using other cyclic monomers.

## Results and Discussion

### Catalyst Design and Characterization

Organic acids are at the forefront among the different families of organocatalysts because of their ability to promote ROP without compromising the control over the polymerization of lactones. However, the use of organic acids in the melt is largely unexplored, mainly because of the poor thermal stability of such compounds at elevated temperatures. Previous results from our group have established that, in contrast with the poor thermal stability of most organic acids, acid–base salts based on 1,5,7-triazabicyclo[4.4.0]dec-5-ene (TBD) and MSA exhibit high thermal stability.^17,30^ On the basis of this previous work, we hypothesized that if a thermally stable catalyst with acid character can be designed, controlled polymerization of lactones could be performed at elevated temperatures. In our search for a thermally stable organocatalyst, DMAP was of particular interest as it is a common choice for ROP because of its commercial availability and relatively low price. However, the poor stability of the catalyst (DMAP or basic mixtures based on DMAP) and the lack of control over the PLLA obtained with such catalysts at elevated temperatures are two important limitations.^31^ As DMAP possesses two different nitrogen atoms on its structure, we initially hypothesized that this catalyst will enable the formation of a thermally stable adduct with two acidic molecules and could promote the ROP of L-LA in a controlled manner.

**Figure 1:**
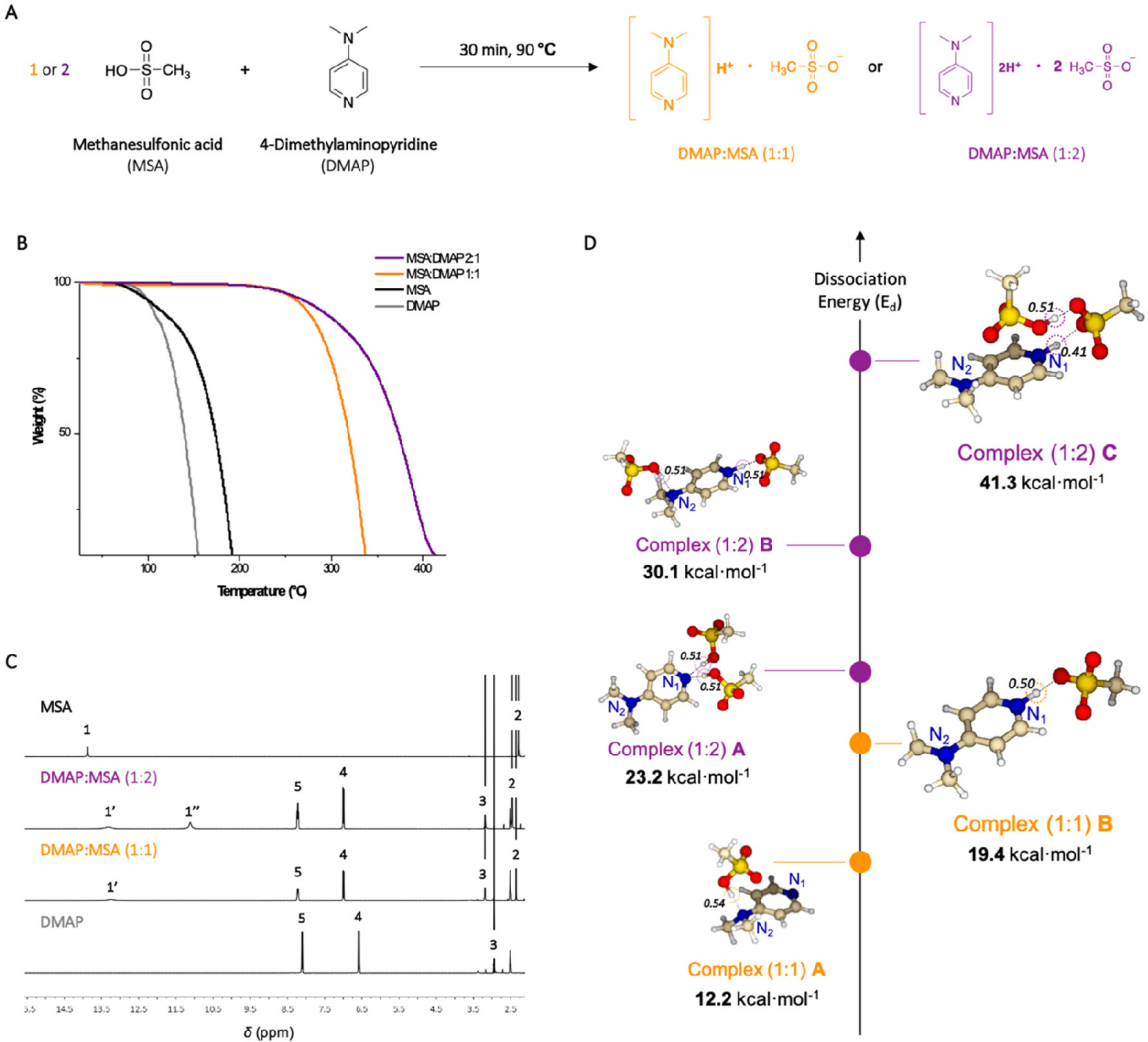
(A) Catalyst synthesis for the nonstoichiometric mixture DMAP:MSA (1:2). (B) Thermogravimetric analysis for MSA, DMAP:MSA (1:2), DMAP:MSA (1:1), and DMAP. (C) ^1^H NMR spectra in DMSO-*d*_6_ (400 MHz, 298 K). (D) DFT optimized geometries for DMAP:MSA (1:2) and DMAP:MSA (1:1) complexes.

DMAP:MSA (1:2) was prepared by mixing MSA and DMAP in the corresponding molar ratio, i.e., 1:2, at 90 °C until a white homogeneous solid was obtained (Figure 1A). For comparison, DMAP:MSA (1:1) was also synthesized following the same procedure. The thermal stabilities of both complexes were then studied by carrying out thermogravimetric analyses (TGA). Both complexes showed much higher thermal stability than the lone acid and base (Figure 1B). Surprisingly, the results also demonstrated that the nonstoichiometric mixture DMAP:MSA (1:2) was stable up to 250 °C, which was higher than DMAP:MSA (1:1) (Figure 1B). This result revealed for the first time a nonstoichiometric mixture resisting higher temperatures than the stoichiometric equivalent.

To demonstrate the complex formation resulting from the proton exchange reaction, DMAP:MSA mixtures were characterized in DMSO-*d*_6_ by ^1^H NMR spectroscopy and compared with the lone components, MSA and DMAP. The recorded spectra for individual MSA and DMAP show the characteristic signal of the MSA acidic proton as a sharp resonance at δ = 14.16 ppm while the characteristic signals for the aromatic ring of DMAP are encountered at δ = 8.09 and 6.59 ppm (Figure 1C). In contrast, in the ^1^H NMR spectra of both the stoichiometric and the nonstoichiometric mixtures, these two signals shift from their initial position to δ = 8.22 and 6.98 ppm, and the signal corresponding to the methyl protons shifts from δ = 2.95 ppm to δ = 3.16 ppm. For DMAP:MSA (1:1), the acidic proton of MSA shifts to a lower value, i.e., δ = 13.26 ppm, while for DMAP:MSA (1:2) two signals corresponding to the acidic protons of the two molecules of MSA are positioned at δ = 13.15 and 11.11 ppm. This demonstrates the formation of a protic ionic salt through proton transfer from MSA to DMAP for both mixtures.

^15^N NMR spectroscopy was also employed to elucidate which type of nitrogen–hydrogen bond was formed (Figure S1). In the recorded spectra, for both DMAP:MSA mixtures, signals attributed to N_1_ in the aromatic ring (δ = 275.68 ppm) and N_2_ on the amine group N(CH_3_)_2_ (δ = 149.61 ppm) are shifted to lower field upon protonation, with N_1_ shifted more when considering the lone DMAP signals. This result hints that N_1_ remains the only nitrogen protonated for both acid–base mixtures. In addition, the weak intensity of the amine group probably results from the extremely long relaxation time of the unprotonated nitrogen site.

To shed some light on this observation and to understand the nature of the DMAP:MSA complexes and the increased stability of the acidic mixture (1:2), DFT calculations were performed by using the Gaussian 16 suite program.^32^ For DMAP:MSA (1:1), the proton transfer from MSA to DMAP could occur toward the nitrogen of the aromatic ring (N_1_) or to the nitrogen linked to the methyl groups (N_2_). DFT optimized structures demonstrate that the complex formed through a proton exchange with N_1_ is more stable (**complex (1:1)B**, dissociation energy (*E*_d_) = 19.4 kcal mol^–1^) when compared to the complex obtained from the proton exchange occurring with N_2_ (**complex (1:1)A**, *E*_d_ = 12.2 kcal mol^–1^) (Figure 1D, in orange). The optimized complex corresponding to DMAP:MSA (1:2) demonstrates even higher stability than DMAP:MSA (1:1), in agreement with the TGA results. The most stable structure shows a first proton transfer between N_1_ and one molecule of MSA and a second proton transfer between the two molecules of MSA (**complex (1:2)C**, *E*_d_ = 41.3 kcal mol^–1^) (Figure 1D, in purple). These results can be correlated to the signals previously observed in the ^1^H NMR spectra of DMAP:MSA (1:2). The structure of **complex (1:2)C** is in agreement with the two distinct resonances observed for the acidic protons of MSA in the ^1^H NMR spectra, including one shifted to the lower frequencies. Mulliken charges extracted from the DFT calculations for all complexes confirm such conclusions (Figure S2).

### Catalyst Evaluation for the Ring-Opening Polymerization of l-Lactide

**Scheme 1:**
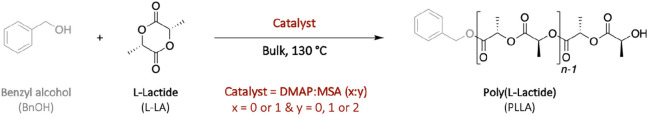
Ring-Opening Polymerization of l-Lactide, in Bulk at 130 °C, Initiated with Benzyl Alcohol Using Different Catalysts

Taking advantage of the high thermal stability of DMAP:MSA mixtures, both the stoichiometric and the acidic complexes were explored as catalysts for the ROP of L-LA into PLLA. The polymerizations were performed in bulk, at 130 °C, with benzyl alcohol (BnOH) as initiator (DP_tot_ = [L-LA]_0_/[BnOH]_0_ = 100) (Scheme 1). Pristine MSA and DMAP were also used as control experiments (Table 1, entries 1–4).

**Table 1:** Conditions and Results for the Ring-Opening Polymerization of l-Lactide in Bulk at 130 °C, Initiated with Benzyl Alcohol and Using Different Catalysts with DP_tot_ = 100

entry	[DMAP]:[MSA]	time (h)	conv (%)^a^	*M*_n,theo_ (g mol^–1^)^b^	*M*_n,NMR_ (g mol^–1^)^a^	*M*_n,SEC_ (g mol^–1^)^c^	*Đ*^c^	*T*_m_ (°C)
1	0:1	2	20					e
2	1:0	3	96	13900	12000	13000	1.4	e
3	1:1	2	99	14400	14580	15000	1.2	e
4	1:2	15	97	14100	13720	15700	1.2	149.6
5^d^	1:1	1	98	7200	6500	9000	1.2	e
6^d^	1:2	4	96	7000	6600	8200	1.2	e

a Calculated by ^1^H NMR spectroscopy.

b Calculated from the molar mass of l-lactide (144.12 g mol^–1^) × conversion × [the initial monomer]/[initiator ratio] + the molar mass of the initiator.

c Determined by SEC in THF with polystyrene standards and correction factors.

d Reactions performed with a degree of polymerization of 50 (DP_tot_ = 50).

e The rest of the samples were completely amorphous.

^1^H NMR spectroscopy was employed to monitor the reactions and characterize the resulting polymers (Figure S3). The disappearance of the characteristic signals of the methine protons of the L-LA monomer at δ = 5.04 ppm and the concomitant appearance of these methine protons in the polymer chain at δ = 5.17 ppm permit to calculate the conversion. When using MSA as catalyst, we achieved only 20% conversion after 2 h, and brownish color was observed, suggesting catalyst degradation. In contrast, DMAP as catalyst resulted in 96% of monomer conversion in 3 h, but the yellow color of the resulting polymer also indicates poor thermal stability. DMAP:MSA (1:1) showed the best catalytic efficiency with 99% of monomer conversion after only 2 h, the resulting polymer being transparent. Similarly, the polymer obtained through the reaction catalyzed by DMAP:MSA (1:2) was colorless, but the reaction was slower, reaching 97% conversion after 15 h.

The molecular weight (*M*_n_) of each PLLA was determined by the integration of the signal attributed to the repeating methine protons of the lactidyl sequence at δ = 5.17 ppm and the aromatic signals of the BnOH initiatior at δ = 7.34 ppm corresponding to the chain end of the polymer. The molecular weights obtained are very similar (from 12000 to 14580 g mol^–1^) and are in good agreement with the theoretical values (Table 1). This result indicates that the ROP of L-LA was strictly initiated by BnOH and attests of the efficiency of the catalysts. Moreover, size exclusion chromatography (SEC) analyses revealed low dispersity, especially for the acid–base mixtures where values of 1.2 were obtained.

**Figure 2:**
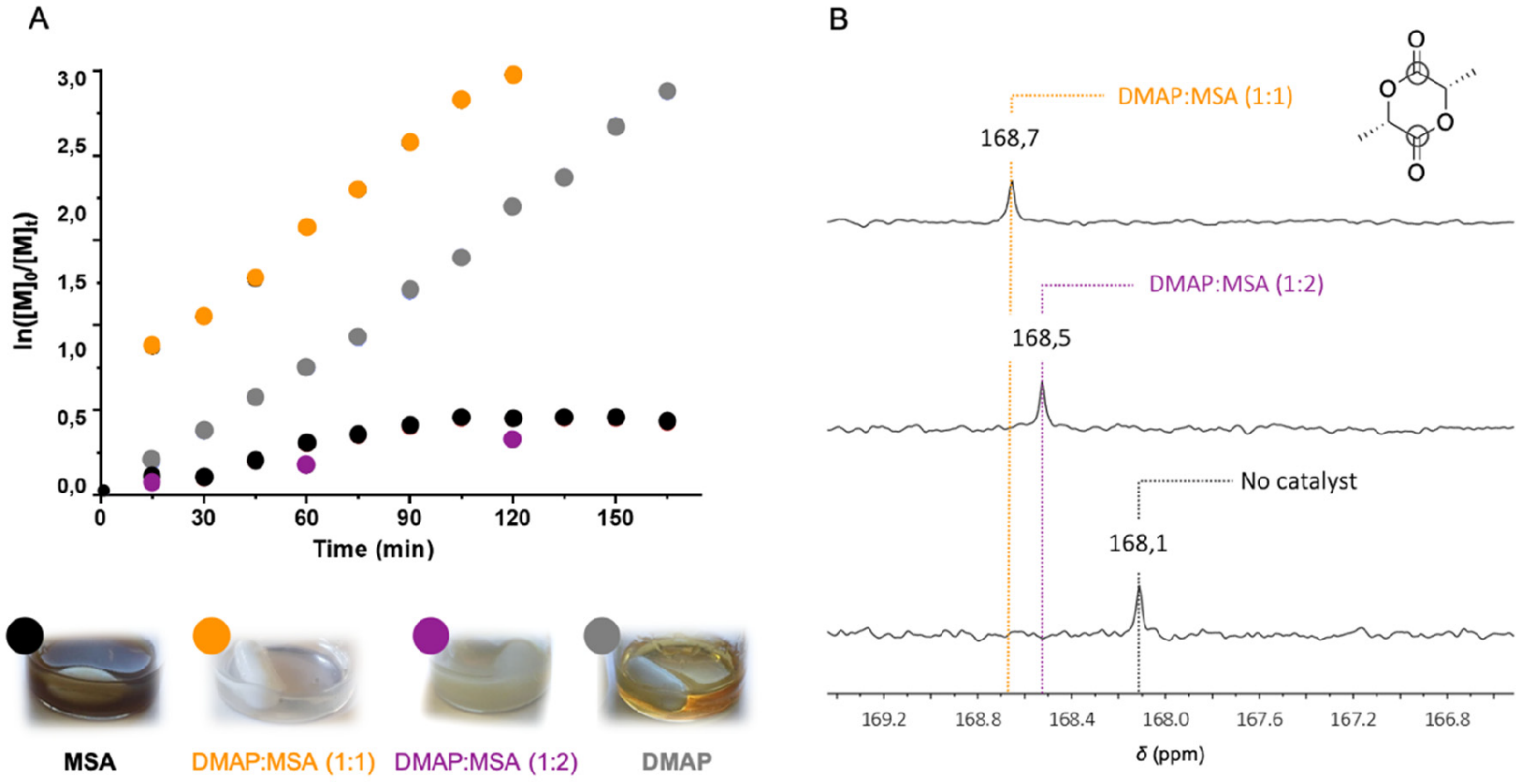
(A) Kinetics of the ring-opening polymerization of l-lactide in bulk at 130 °C with MSA, DMAP:MSA (1:1), DMAP:MSA (1:2), and DMAP. (B) ^13^C NMR spectra in CDCl_3_ (400 MHz, 298 K) of the complexes formed by l-lactide and DMAP:MSA mixture catalysts.

First-order kinetics were plotted for each catalyst for the polymerization performed with an initial [L-LA]_0_/[BnOH]_0_/[cat]_0_ of 100/1/1 (Figure 2A). These plots showed a linear tendency, suggesting a living polymerization. To confirm this, a study of the evolution of the molecular weight of PLLA was performed for a targeted degree of polymerization of 50 catalyzed by both DMAP:MSA (1:1) and DMAP:MSA (1:2) (Table 1, entries 5 and 6). In both cases the evolution of *M*_n,SEC_ was linear, independent of the catalytic system (Figures S4). Finally, to confirm end-group fidelity, BnOH was substituted by a fluorescent initiator, 4-pyrenebutanol, for both DMAP:MSA (1:1) and (1:2) catalyzed ROP of L-LA. SEC characterization with both refractive index (RI) and UV detection was then performed on the resulting polymers. The UV–vis and RI SEC traces for PLLA are perfectly overlaid, indicating that pyrene moieties are end-capping PLLA chains, thus confirming the absence of transesterification reactions (Figures S5).

To understand the differences in polymerization rate when using DMAP:MSA (1:2) and DMAP:MSA (1:1), the interaction between the monomer and the catalysts was investigated by ^13^C NMR spectroscopy. Mixtures of equimolar amounts of L-LA and the catalysts, i.e., DMAP:MSA (1:1) and DMAP:MSA (1:2), were analyzed by ^13^C NMR in CDCl_3_ (Figure 2B). In the carbonyl region, the resonance of the carbonyl of L-LA is significantly affected by the presence of both catalysts. Interestingly, this effect is more pronounced when L-LA is in the presence of 1 mol equiv of DMAP:MSA (1:1) catalyst. This higher shielding effect confirms that the carbonyl is more activated by DMAP:MSA (1:1), thus, favoring the nucleophilic attack and explaining why the polymerization is faster, while compared with the (1:2) mixture.

### Promoting Control over the Ring-Opening Polymerization of l-Lactide

**Figure 3:**
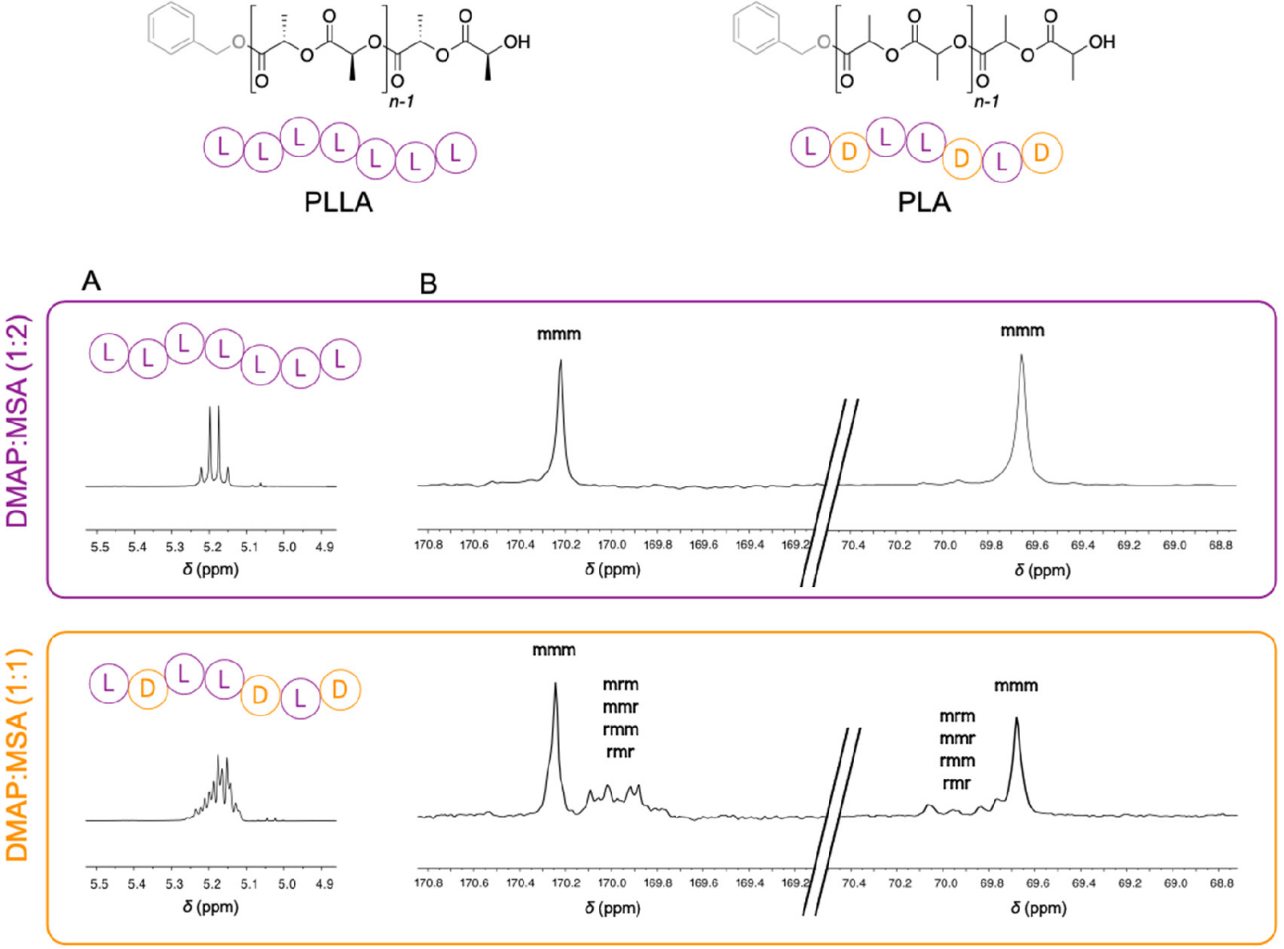
(A) ^1^H NMR spectra and (B) ^13^C NMR spectra, in DMSO-*d*_6_ (400 MHz, 298 K), for the polymers resulting from the ring-opening polymerization of l-lactide catalyzed by DMAP:MSA (1:2) (in purple) and DMAP:MSA (1:1) (in orange).

One of the remaining challenges in the ROP of L-LA is to avoid side reactions such as transesterification and epimerization which provide nonstereoregular PLA. The control of the polymer microstructure is of great importance since it affects the mechanical and thermal properties of the obtained PLLA.^25,26^ To determine if the catalysts can promote the ROP of L-LA at 130 °C in a controlled manner, the purified polymers obtained with DMAP:MSA (1:2) and DMAP:MSA (1:1) were characterized by ^1^H and ^13^C NMR spectroscopy techniques. In a stereoregular PLLA, the characteristic signal of the lactidyl protons is a quadruplet in the methine region of the ^1^H NMR spectrum (δ = 5.1–5.3 ppm). For the polymer obtained with the acidic (1:2) catalyst, a well-defined quadruplet signal can be observed at δ = 5.18–5.20 ppm while the corresponding signal in the spectra of the polymer obtained with the stoichiometric mixture is an undefined multiplet (Figure 3A). This first characterization suggests that the DMAP:MSA (1:2) catalyst leads to a stereoregular PLLA while the (1:1) mixture apparently promotes defects during the polymerization. A similar conclusion can be drawn from the analysis of the NMR spectrum of the polymer synthesized with DMAP as catalyst (Figure S6).

The stereoregular structure of the PLLA can also be confirmed by analysis of the ^13^C NMR spectra. The PLLA resulting from the reaction with DMAP:MSA (1:2) exhibits a lone singlet signal at δ = 170.3 ppm and δ = 69.6 ppm attributed to the *mmm* tetrad (Figure 3B). In contrast, in the spectra of the polymer obtained with DMAP:MSA (1:1), extra undefined signals attributed to different tetrads are also observed at δ = 169.6–170.2 ppm and δ = 69.6–70.2 ppm, suggesting the presence of defects along the chain (Figure S7).

As mentioned before, the stereoregularity of the PLLA impacts its physical properties. Thus, the analysis of the thermal properties of the polymer can offer an insight into the differences in the degree of stereoregularity of PLA/PLLA polymers, which range from amorphous to semicrystalline.^33,34^ Samples obtained from DMAP:MSA (1:1) and (1:2) catalysts were analyzed by differential scanning calorimetry (DSC) (Figure S8). The resulting curves demonstrate that semicrystalline PLLA was obtained with DMAP:MSA (1:2), with a *T*_m_ of 149.6 °C, while amorphous PLA was obtained with DMAP:MSA (1:1).

Finally, the two polymers were analyzed by ^13^C NMR to calculate the stereoregularity (Figure S9). The PLLA prepared with the acidic DMAP:MSA (1:2) presents a L- to D-isomer ratio of *P*_m_ = 0.98 while the sample obtained from the stoichiometric mixture has a ratio of *P*_m_ = 0.52. Taking into account that a very minor amount of epimerization can detrimentally affect the PLLA stereoregularity, this result undoubtedly confirms that DMAP:MSA (1:2) promotes stereoregular ROP while not DMAP:MSA (1:1).

### Polymerization Mechanism Investigation

**Scheme 2:**
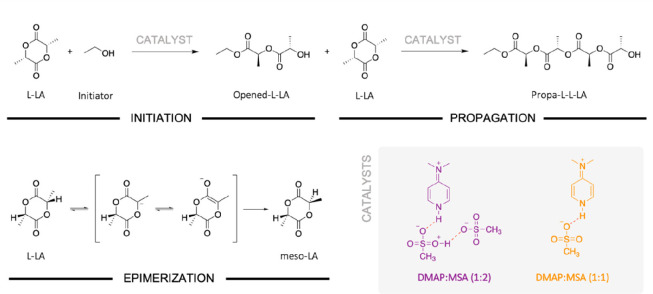
Model Reaction for the Initiation, Propagation, and Epimerization for the Ring-Opening Polymerization of l-Lactide (L-LA)

To explain such differences in kinetics and stereoregularity between DMAP:MSA (1:2) and (1:1), mechanistic investigations were performed by means of quantum chemical calculations. Computational investigations were performed with the ωB97XD functional in conjunction with the 6-31+G(d,p) basis set for all atoms for geometric optimization (see the Supporting Information for computational details). Initiation, propagation, and epimerization for the ROP of L-LA were investigated by comparing the acidic and the stoichiometric systems. The studies available in the open literature that provide mechanistic insights into ROP of L-LA catalyzed by acidic catalysts have all observed a bifunctional mechanism.^35−37^ Thus, this mechanism was explored to compare DMAP:MSA (1:1) with DMAP:MSA (1:2). To perform the calculations in a reasonable amount of time, BnOH, which is employed as initiator experimentally, was modeled by a molecule of ethanol (Scheme 2).

Initiation involves the opening of the cyclic monomer to yield an initial propagating chain of the polymer, i.e., **Opened-L-LA**. Then the propagation step involves the opening of a second molecule of L-LA by a nucleophilic attack of the linear **Opened-L-LA** to yield **Propa-L-L-LA**, corresponding to the propagating chain composed of two opened monomeric units. Both steps, the initiation and the propagation, take place through two transition states (TSs). This includes, first, the nucleophilic attack of the hydroxyl of the initiator or the propagating chain hydroxyl on L-LA (**TS I1** or **TS I2**) and, second, the ring-opening of the cyclic ester (**TS P1** and **TS P2**).

**Scheme 3:**
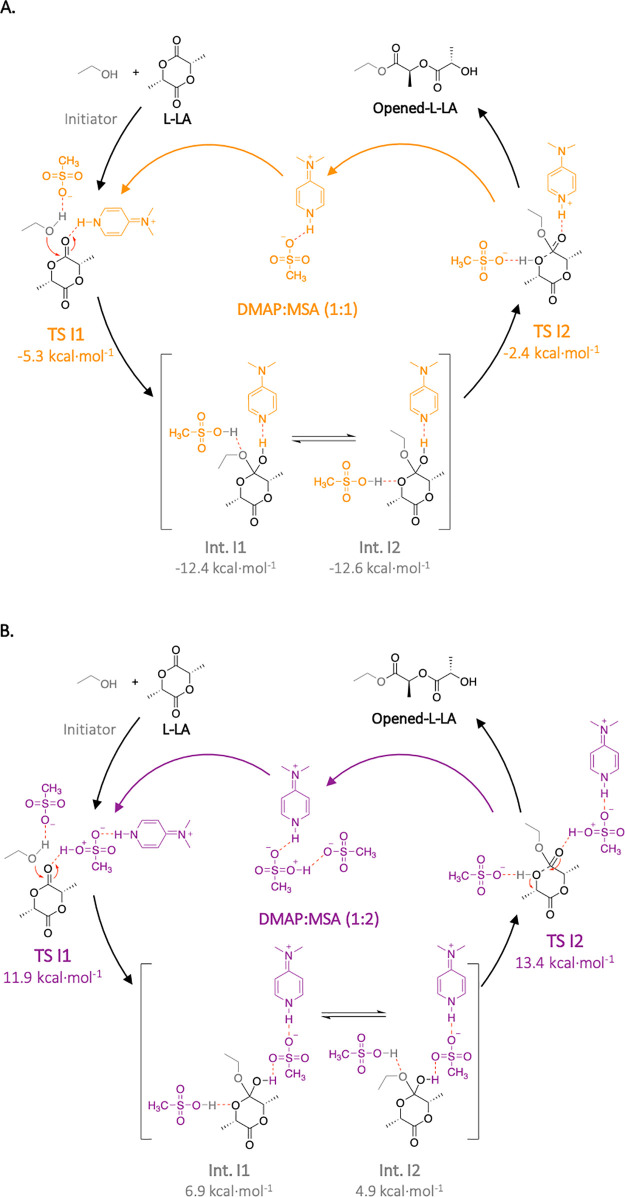
Proposed Mechanisms for the Initiation of the Ring-Opening Polymerization of l-Lactide with Energetic Stationary Levels Calculated at the ωB97XD/6-311++G(2df,2p) Level of Theory Catalyzed by (A) DMAP:MSA (1:1) and (B) DMAP:MSA (1:2)

For initiation and propagation, the first transition states for both catalysts are undeniably limiting steps with energetic barriers of more than 20 kcal mol^–1^, while the second transitions states have an energy barrier between 8 and 10 kcal mol^–1^ (Figure S10). For each catalyst, the initiation and propagation steps are very similar, which is in agreement with the first-order kinetics found experimentally. Comparing DMAP:MSA (1:2) and (1:1), the distinct energetic levels for both pathways suggest very different kinetics. While all transition states for the reaction catalyzed by the stoichiometric mixture are below zero (Scheme 3A), transition states for DMAP:MSA (1:2) are encountered at more than 11.9 kcal mol^–1^ (Scheme 3B). As a result of the negative values, the reaction catalyzed by DMAP:MSA (1:1) is fast. Moreover, the final complexes for initiation and propagation are also negative, −21,7 and −8.2 kcal mol^–1^, respectively, suggesting a thermodynamically driven reaction.

In contrast to this behavior, for DMAP:MSA (1:2), apart from initial complexes, transitions states and stationary levels are all positioned above 0, including final complexes at 5.6 and 6.8 kcal mol^–1^ for initiation and propagation, respectively. This energetic difference is in agreement with what was previously observed experimentally, in which the polymerization catalyzed by DMAP:MSA (1:1) was 7 times faster than when DMAP:MSA (1:2) was employed.

However, no significant difference can be observed for the energetic barriers (in the case of **TS I1** for example, 21.5 kcal mol^–1^ for the acidic mixture and 23.6 kcal mol^–1^ for DMAP:MSA (1:1)). Here, it has to be taken into account that the computational investigations have been done in the gas phase while the reaction is experimentally performed in bulk, which significantly impacts equilibria along the reaction, notably the complexes formed by the isolated molecules (**Complex I1, I2, P1**, and **P2**). If no solvent model was employed to better model the reaction, it is because the solvent models largely rely on the permittivity of the solvent, which is not an available data for L-LA.

**Figure 4:**
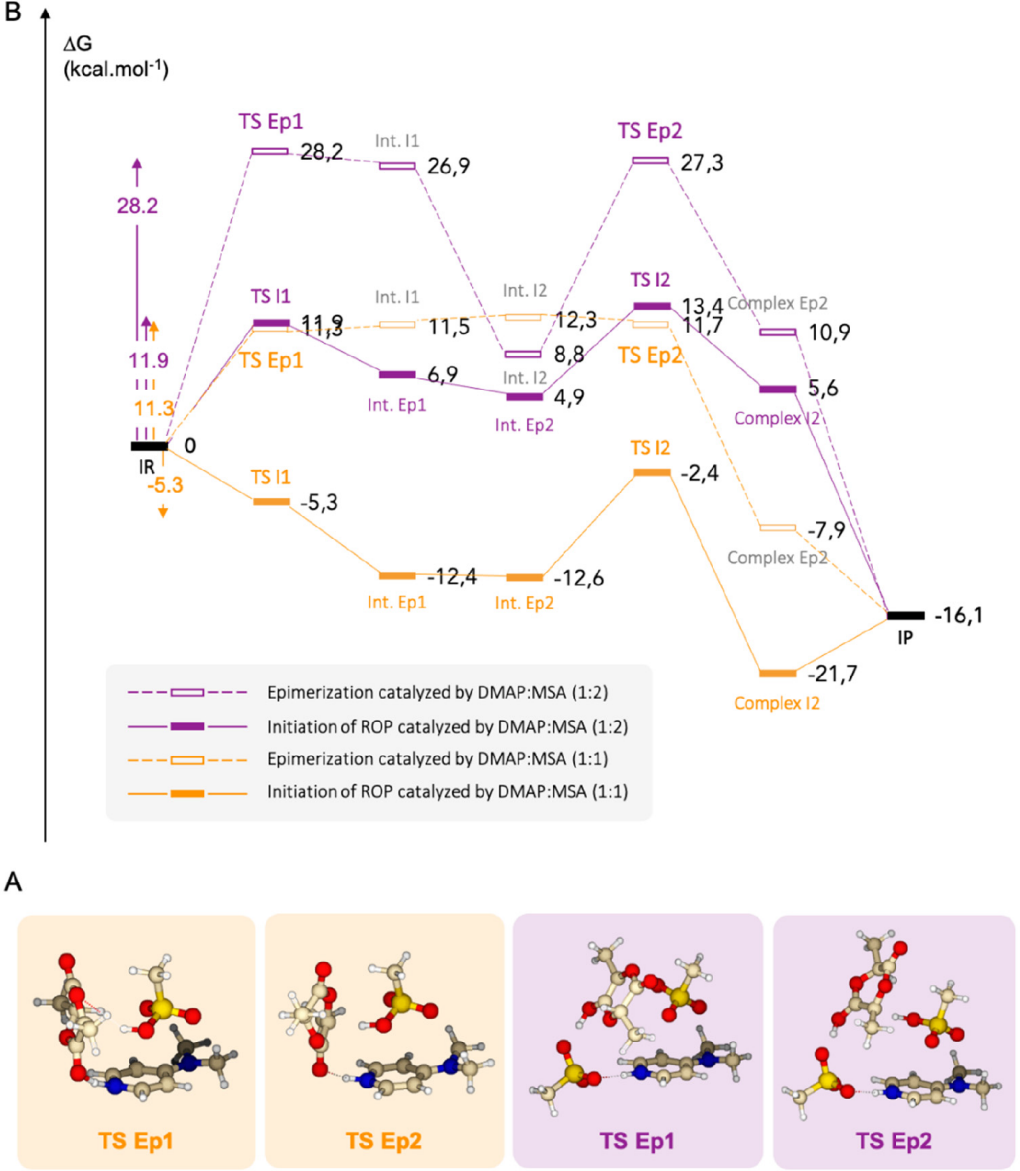
(A) DFT-computed pathways for the epimerization (dashed lines) and the propagation (plain line) mediated by DMAP:MSA (1:1) (in orange) and DMAP:MSA (1:2) (in purple). (B) Associated isolated structures for transition states. Color code: gray, C; white, H; red, O; blue, N; yellow, S. Calculations were performed at the ωB97XD/6-311++G(2df,2p) level of theory.

Although the polymerization is faster when employing the stoichiometric mixture, the stereocontrol offered by the acidic catalyst provides a tremendous advantage to this system. Experimental results suggested that the good control over the reaction catalyzed by DMAP:MSA (1:2) is due to a limited extent of epimerization as compared to when DMAP:MSA (1:1) was used. Thus, the epimerization of L-LA, i.e., the transformation of L-LA into meso-LA, was also investigated. The results demonstrate that while the epimerization mediated by the stoichiometric mixture only requires 11.3 kcal mol^–1^, the same reaction catalyzed by the acidic DMAP:MSA (1:2) requires 28.2 kcal mol^–1^ (Figure 4).

In the case of DMAP:MSA (1:1), although the energy demanded for the epimerization is higher than the energetic barrier to overcome **TS I1** (when the isolated reagents are considered as starting stationary points), it is similar to the energetic barrier for the initiation of the reaction catalyzed by the acidic mixture —11.3 and 11.9 kcal mol^–1^, respectively. This result suggests that in the case of reactions catalyzed by DMAP:MSA (1:1), although epimerization is unfavorable when compared to initiation, it is still a feasible reaction, which is in agreement with the 18% of epimerization found experimentally. On the contrary, the high energy required for the epimerization of L-LA mediated by DMAP:MSA (1:2) (28.2 kcal mol^–1^) as compared to the initiation step (11.9 kcal mol^–1^) indicates that it is a highly improbable reaction, in agreement with the experimental observations.

### Investigating the Use of DMAP:MSA (1:2) at Industrially Relevant Conditions

Industrially, bulk polymerization of L-LA is typically performed between 150 and 180 °C in the presence of tin octoate, the catalyst which has presented the best performance to date, i.e., reaching high molecular weights with minimum side reactions. Because the polymerization is pseudoliving, the molecular weight can be controlled up to moderate conversions; until side reactions, particularly intermolecular transesterification significantly broadens the molecular weight distribution. To compare the efficiency of DMAP:MSA (1:2) with the procedure employed industrially, the polymerization temperature was raised from 130 °C to 150 and 180 °C for a targeted degree of polymerization of 100 (Table 2, entries 7–9). The catalyst concentration was adjusted to obtain high conversions at relatively low reaction times (6 h). The theoretical and experimental molecular weights are very similar, independent of the temperature employed, and SEC analyses revealed low dispersity values for all reactions (*Đ* = 1.2). The thermal properties and microstructures observed by DSC and ^13^C NMR spectroscopy demonstrate similar results for the PLLA synthesized in all three reactions, expanding the potential of DMAP:MSA nonstoichiometric mixture to operate in a controlled manner under industrially relevant conditions.

**Table 2:** Results and Conditions for the ROP of L-LA in Bulk Initiated with Benzyl Alcohol

entry	[BnOH]: [cat.]:[L-LA]	temp (°C)	time (h)	conv (%)^a^	*M*_n,theo_ (g mol^–1^)^b^	*M*_n,NMR_ (g mol^–1^)^a^	*M*_n,SEC_ (g mol^–1^)^c^	*Đ*^c^	*T*_m_ (°C)	Δ*H* (J/g)
7	1:2:100	130	8	98	14200	14500	14300	1.2		
8	1:2:100	150	6	96	13900	14400	15200	1.2	149.3	39.28
9	1:2:100	180	6	98	14200	14700	15800	1.2		
10	1:2:200	150	14	99	28600	32100	24500	1.2	149.5	35.10
11	1:2:400	150	26	98	56600	56500	40100	1.3	149.9	57.28

a Calculated by ^1^H NMR spectroscopy.

b Calculated from the molar mass of l-lactide (144.12 g mol^–1^) × conversion × [the initial monomer]/[initiator ratio] + the molar mass of the initiator.

c Determined by SEC in THF with polystyrene standards and correction factors.

**Figure 5:**
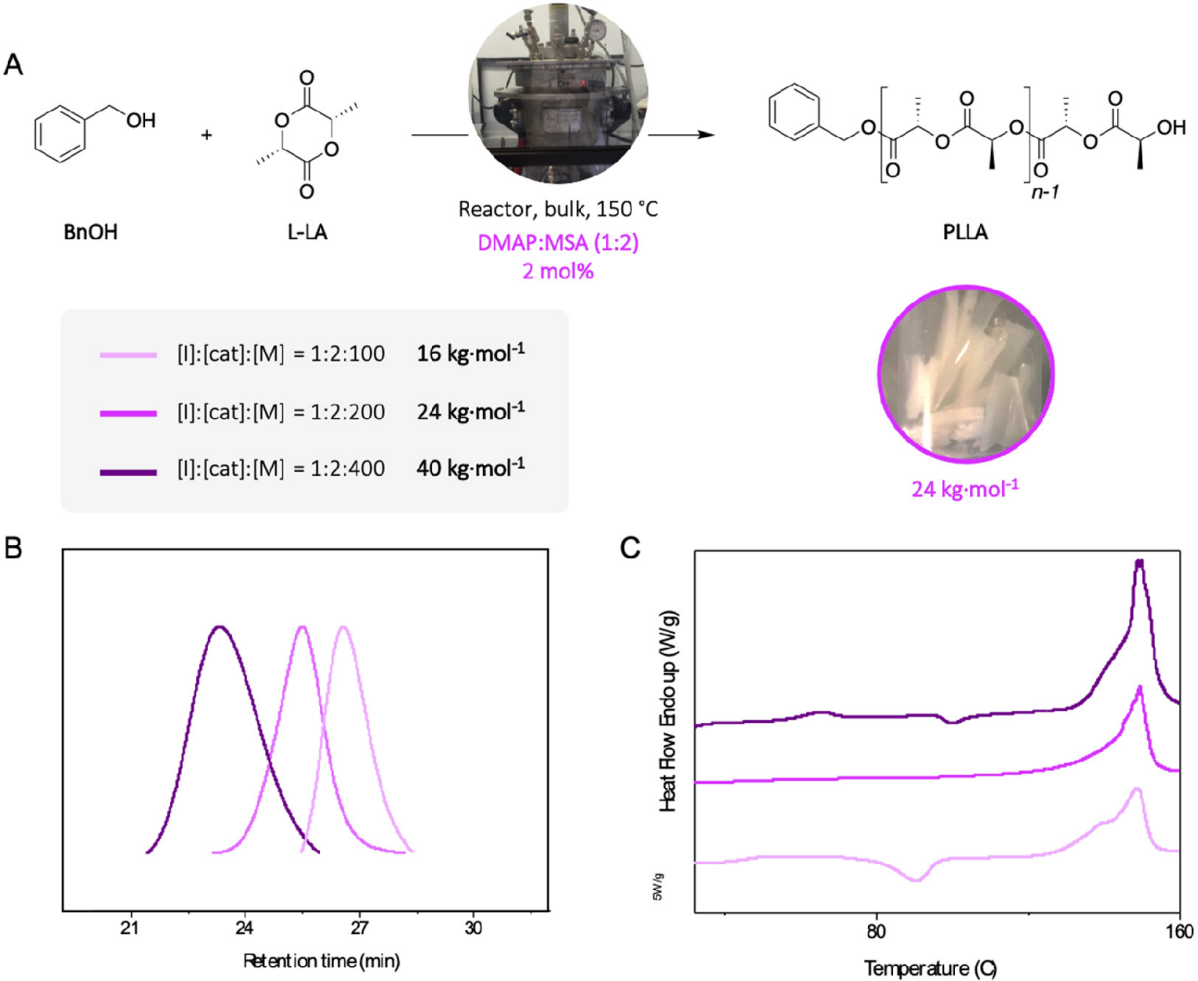
(A) Ring-opening polymerization of l-lactide initiated by benzyl alcohol in a reactor of 5 kg, in bulk and at 150 °C, catalyzed by DMAP:MSA (1:2). (B) SEC traces and (C) DSC cooling scans for different degrees of polymerization for a monomer [M] to initiator [I] ratio of 100, 200, and 400.

Finally, despite the great potential of the DMAP:MSA (1:2) catalyst in the ROP of L-LA at elevated temperatures, one of the remaining challenges is the synthesis of high molecular weights suitable for commercial implementation—above 30 kg mol^–1^. Thus, to reach industrially relevant molecular weights, the monomer-to-initiator ratio was increased from 100 to 200 and 400, with the reaction was performed at 150 °C, in a 5 kg reactor (Figure 5A). The PLLA obtained was white, indicating that the catalyst was not degraded, and molecular weights of *M*_n,NMR_ = 32100 g mol^–1^ and *M*_n,NMR_ = 56500 g mol^–1^ were obtained for targeted degrees of polymerization of 200 and 400, respectively (Table 2, entries 10 and 11). SEC traces of the PLLAs prepared exhibit narrow and symmetrical distributions (Figure 5B). When aiming high molecular weights, the stereoregularity was maintained as shown in the DSC scans, which demonstrates the highly semicrystalline nature of the samples (Figure 5C). In all the cases semicrystalline materials with *T*_m_ values close to 150 °C and Δ*H* values between 35 and 57 J.g^–1^ were obtained.

### Expanding the Scope to the Preparation of Block Copolymers

**Figure 6:**
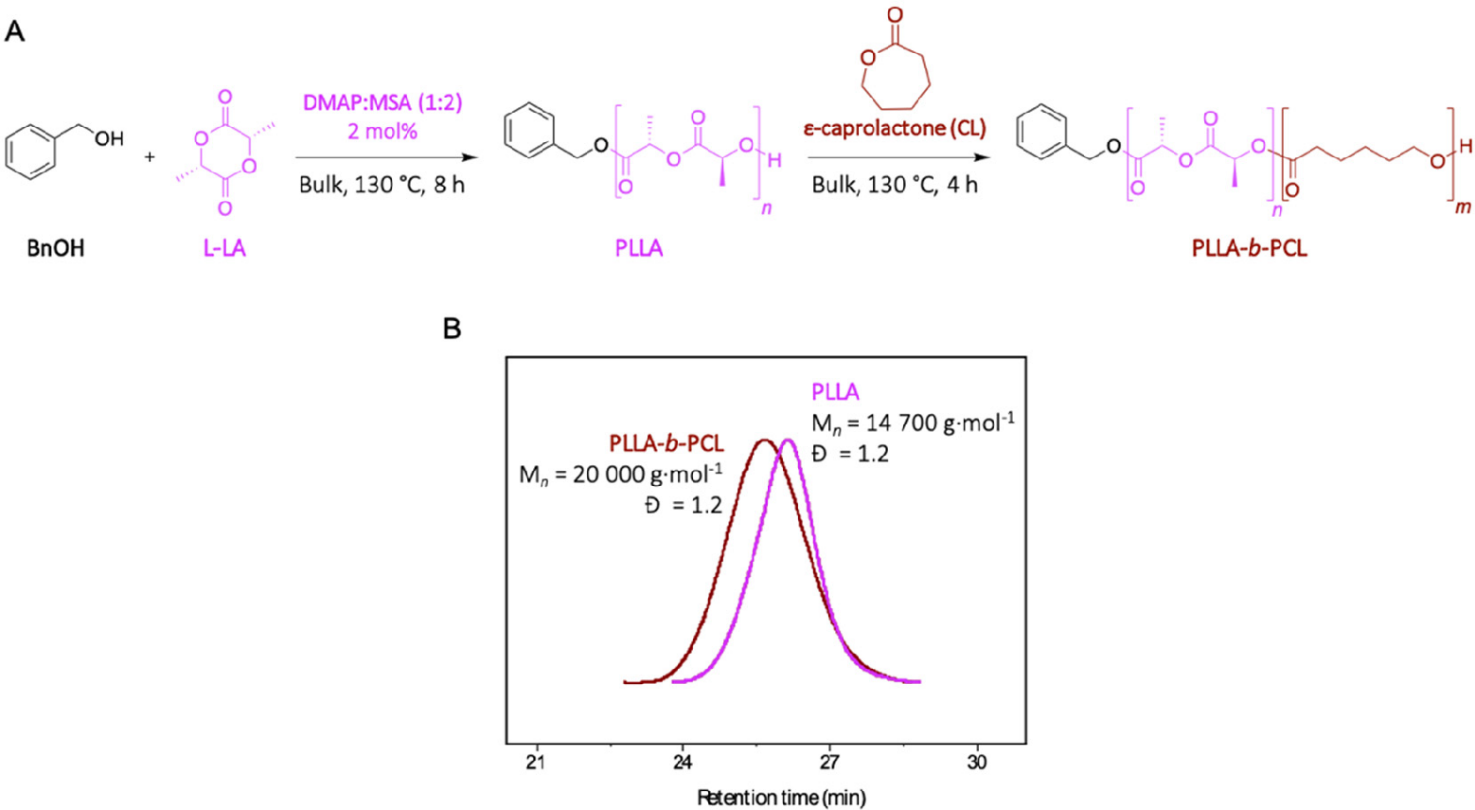
(A) Ring-opening copolymerization of l-lactide and ε-caprolactone initiated with benzyl alcohol, in bulk and at 130 °C, catalyzed by DMAP:MSA (1:2). (B) SEC traces for the polymer before and after addition of ε-caprolactone.

ε-Caprolactone (CL), another common cyclic ester monomer, was polymerized to explore the potential of the DMAP:MSA (1:2) catalytic system. The homopolymerization of CL was successfully performed in bulk at 130 °C, with 2 mol % of catalyst, and the resulting polycaprolactone (PCL) was analyzed by ^1^H NMR spectroscopy and SEC (Figures S11 and S12). Conversion of 94% and a molecular weight of 11100 g mol^–1^ was obtained after 4 h, demonstrating a faster polymerization than for L-LA under the same conditions. The copolymerization of CL and L-LA was finally performed in a two-step, one-pot reaction. The ROP of L-LA was first performed in bulk, at 130 °C, with 2 mol % of catalyst for a targeted degree of polymerization of 100 (Figure 6A). After 8 h, full conversion of L-LA was attained, and 1 equiv of CL—compared to L-LA—was added while the reaction was kept at 130 °C for an additional 4 h.

SEC analyses were performed before and after the addition of the CL monomer. As expected after CL addition, the SEC trace is shifted to higher molecular weights (from 14700 to 20000 g mol^–1^), confirming the copolymer formation while maintaining a low dispersity (Figure 6B). After purification, the analysis of the resulting polymer through ^1^H NMR spectroscopy corroborates that a PLLA-*b*-PCL copolymer with a L-LA-to-CL ratio of 60:40 was obtained (Figure S13). The thermal properties of the copolymer were also analyzed and compared with PLLA and PCL homopolymers (Figure S14).

## Conclusion

In this work, computational and experimental studies were combined to explore the use of an acidic hydrogen-bond-based catalyst synthesized from a mixture of methanesulfonic acid (MSA) and 4-(dimethylamino)pyridine (DMAP). DMAP:MSA (1:2) permits the preparation of stereoregular PLLA through ring-opening polymerization (ROP), in bulk conditions and at elevated temperatures (up to 180 °C), thanks to the positive combination of (1) the thermal stability of acid–base mixtures and (2) the excellent control over the polymer structure and molecular weight distributions of organic acids. The reaction was applied to synthetic procedures relevant for industry, providing further evidence of the good catalyst control of the reaction and resulting in a colorless PLLA of 40 kg mol^–1^. Computational investigations confirmed that in the presence of DMPA:MSA (1:2) epimerization is much less favorable, which confirms the greater performance of the acid-rich complex. This unprecedented performance of an organocatalyst under industrially relevant conditions illustrates a new concept that can be general and could be useful for a wide range of high-temperature reactions. This is particularly important given the increasing demand for the replacement of conventional metal catalysts by organocatalysts.

## Supporting Information Available

Experimental and computational details; optimized Cartesian coordinates; Figures S1–S14 (PDF)
