# Supplementary material for: Stereoretention in the Bulk ROP of l-Lactide Guided by a Thermally Stable Organocatalyst
Source: Macromolecules. 2021 Jun 16;54(13):6214–25. doi: 10.1021/acs.macromol.1c01060 (PMC9171820; doi:10.1021/acs.macromol.1c01060)
Supplement: Supplementary file 1 — ma1c01060_si_001.pdf [file ma1c01060_si_001.pdf]

STEREORETENTION IN THE BULK ROP OF L-LACTIDE GUIDED BY A THERMALLY STABLE ORGANOCATALYST

Andere Basterretxea[1⊥], Elena Gabirondo[1⊥], Coralie Jehanno[1], Haijin Zhu[2], Olivier Coulembier[3], David Mecerreyes[1,4], Haritz Sardon[1]

[1] POLYMAT, University of the Basque Country UPV/EHU, Paseo Manuel de Lardizabal 3, 20018, Donostia–San Sebastian, Spain.

[2]Deakin University Burwood Campus, Institute for Frontier Materials, 221 Burwood Highway, Geelong, Victoria 3125, Australia

[3] Center of Innovation and Research in Materials and Polymers (CIRMAP), Laboratory of Polymeric and Composite Materials, University of Mons, Place du Parc 23, 7000 Mons, Belgium.

[4] IKERBASQUE Basque Foundation for Science, 48009 Bilbao, Spain

⊥ these two authors contribute equally

1. Experimental Section

1.1 Materials

L-Lactide (L-LA, 98%, TCI) was recrystallized three times from toluene and dried under vacuum for two days. ε-Caprolactone (CLO, 99%, Sigma Aldrich), benzyl alcohol (BnOH, 99%, Sigma Aldrich) were dried over CaH2 for 48 hours prior to their distillation under reduced pressure and were stored on molecular sieves and 1-pyrenebutanol (99%, Sigma Aldrich) were also dried under vaccum. Compounds were stored in a glove box (O2 ≤ 1 ppm, H2O ≤ 0.5 ppm). Methanesulfonic acid (MSA, 99%) and 4-(dimethylamino)pyridine (DMAP, 98%), chloroform ($CDCl_3$), methanol (CH_3OH) and the rest of the solvents used on this work were supplied by Sigma-Aldrich and used as received.

1.2 Characterization Techniques

1.2.1 Nuclear Magentic Resonance (NMR) spectroscopy

^1H and ^{13}C Nuclear Magnetic Resonance (NMR) spectroscopy was recorded in a Bruker Avance DPX 300 at 300.16 MHz and at 75.5 MHz of resonance frequency respectively, using deuterated chloroform ($CDCl_3$) or as solvent at room temperature. The conditions are the following ones: (a) For 1H NMR spectroscopy: 10 mg of sample; 3 s acquisition time; 1 s delay time; 8.5 µs pulse; spectral width 5000 Hz and 32 scans. (b) For 13C NMR spectroscopy: 40 mg; 3 s acquisition time; 4 s delay time; 5.5 µs pulse; spectral width 18 800 Hz and more than 10 000 scans.

1.2.2 Size Exclusion Chromatography (SEC)

SEC measurements were performed at 30 °C on an Agilent 1200 system equipped with PLgel 5 μm Guard and PLgel 5 μm MIXED-C columns and a differential refractive index (RI) detector (Optilab Rex, Wyatt). THF was used as eluent at a flow rate of 1 ml/min and calibrated using narrow polystyrene standards ranging from 595 to $3.95 \cdot 10^{-6}$ g mol^{-1} (5th order universal calibration).

1.2.3 Differential Scanning Calorimetry (DSC)

Differential Scanning Calorimeter (DSC-Q2000, TA Instrument Inc., USA) was recorded using 6-8 mg to calculate the glass transition and melting temperatures. First scan was carried out in all cases to eliminate the thermal history of the sample and later on the samples were analyzed between 25 °C to 160 °C at 20 °C·min^{-1}. The temperatures were calculated from the second heating run.

1.2.4 Thermogravimetric analyses (TGA)

TGA analysis were performed under nitrogen (20ml·min^{-1} flow rate) using a TGA Discovery (TA Instruments). The procedure is composed of a first equilibration at 100ºC for 20 min and then heated at a rate of 10ºC·min^{-1}, in the range from 25ºC to 800ºC.

1.2.5 Computational details

All calculations were carried out employing the Gaussian 16 suite of programs. Geometry optimizations were performed with the ωB97XD density functional and the 6-31+G(d,p) basis set. Frequency optimizations at the same level of theory confirmed that the optimized structures were minima (zero imaginary frequencies) or transition states (one imaginary frequency) and then used to evaluate the ZPVE and the thermal vibrational corrections at T = 298 K. The electronic energy was refined by single-point energy calculations at the ωB97XD/6-311++G(2df,2p) level of theory. The IRC method has been used to verify that the obtained transition states were connected to the desired minima.

1.3 Methods

1.3.1 General procedure of the preparation of the catalyst DMAP:MSA

In a glove box, different dual catalysts were prepared by mixing methanesulfonic acid (MSA) (1.38 10^{-4} mol, 0.013 g, 9 μl) and 4-dimethylaminopyridine (DMAP) (1.38 10^{-4} mol, 0.017 g) for the mixture 1:1 while methanesulfonic acid (MSA) and (2.76 10^{-4} mol, 0.026 g, 18 μl) and 4-dimethylaminopyridine (DMAP) (1.38 10^{-4} mol, 0.017 g) were used for the preparation of the mixture 1:2. Afterwards, the mixtures were thermally treated at 90 °C over 30 minutes under

stirring until complete formation of homogeneous and transparent liquid solution or salt. The good formation of those complexes was attested by 1H NMR spectroscopy prior their use.

1.3.2 General procedure of the Ring-Opening Polymerization of L-lactide and ε-caprolactone catalyzed by the DMAP:MSA mixtures

In a glove box, previously dried vials were charged with LLA (1 g), the DMAP:MSA catalyst (1:1, 1:2) and a stir bar. Then the initiator (BnOH,) was added via a 5 or 10 μL syringe. The sealed vials were immersed in a pre-heated oil bath at the desired temperature (130 °C–180 °C). The conversion of the monomer was monitored by ^{1}H NMR spectroscopy with solution of $CDCl_3$ until reached at least 95%. The reaction was stopped by cooling down the medium at room temperature. For the purification, the crude polymer was dissolved in chloroform and precipitated in cold methanol. The resulted PLLA were filtrated and dried under vacuum at RT for 24 h before their characterization. The same procedure was used in the ROP of ε -caprolactone.

1.3.3 Diblock copolymerization of L-lactide and ε-caprolactone

In a glove box, LLA (0.5 g) the DMAP:MSA 1:2 and a stir bar were added to a dry vial .Then, the BnOH initiator was added via a 5 or 10 μL syringe and the sealed vial was immersed, out of the glovebox, in pre-heated oil bath thermostatized at 130°C. The LLA conversion of LLA was followed by ^{1}H NMR (in $CDCl_3$) When the conversion value was of 90% (~8 hours), the polymerization medium was cooled down at RT and the CLO (0.4 g) was added in the vial by the use of a glove box. Very quickly, the vial was re-immersed at 130°C. After 4 h, the copolymer (PLLA-b-PCLO) was isolated by dissolving it in chloroform prior its precipitation in cold methanol. After decantation, the copolymer was filtered out and dried under vacuum.

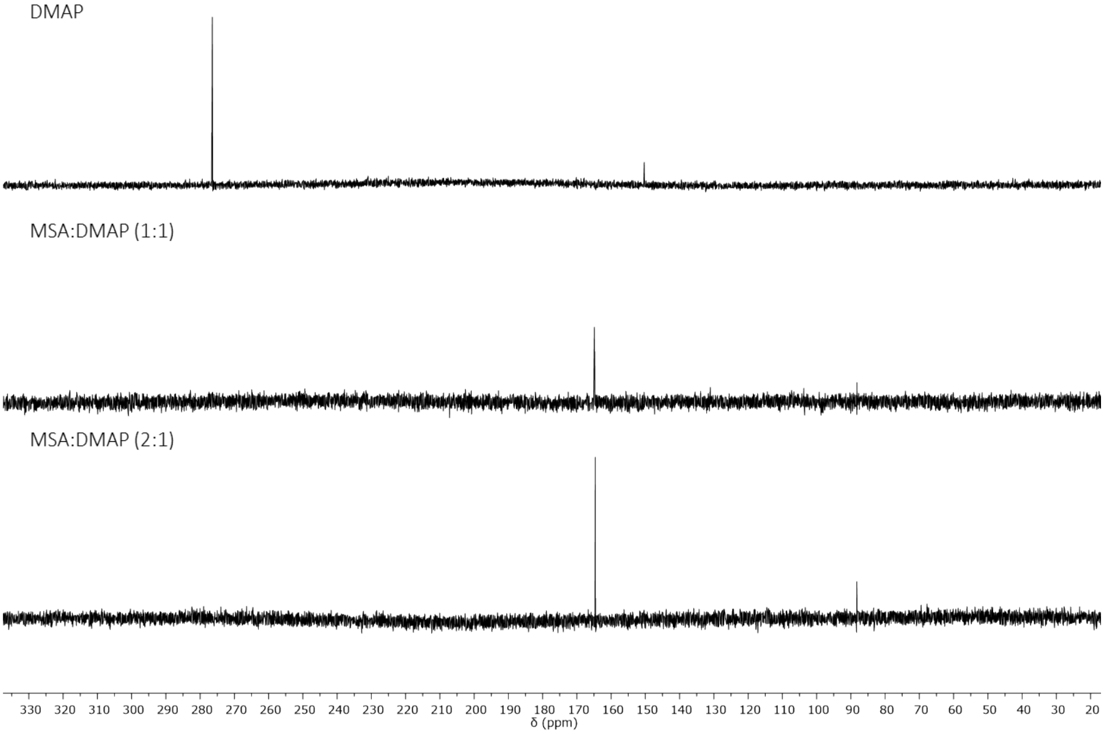

Figure S1. *^{15}N NMR spectra of the catalyst DMAP, DMAP:MSA (1:1) and DMAP:MSA (1:2).*

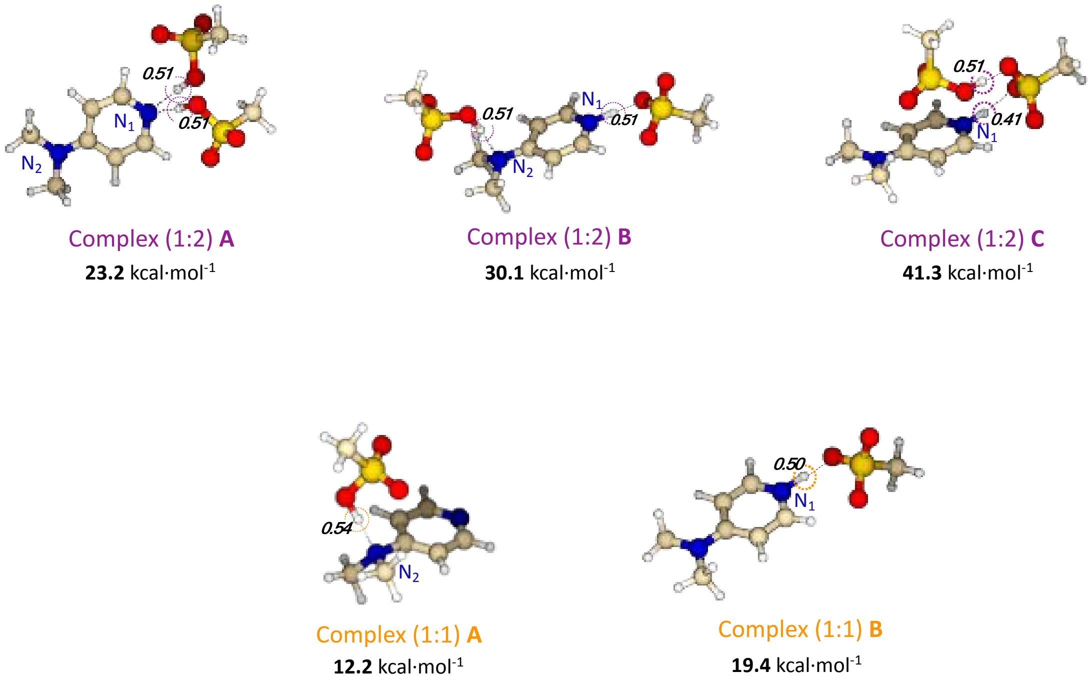

Figure S2. *Optimized structures for DMAP:MSA (1:1) (in orange) and DMAP:MSA (1:2) (in purple). Mulliken charges were extracted from DFT calculations and the resulting partial charges*

map on nitrogen atoms foreach complex demonstrate the unbalanced repartition for the less stable complexes, i.e. ***complex (1:1) A***, ***complex (1:2) A*** *and* ***complex (1:2) B.***

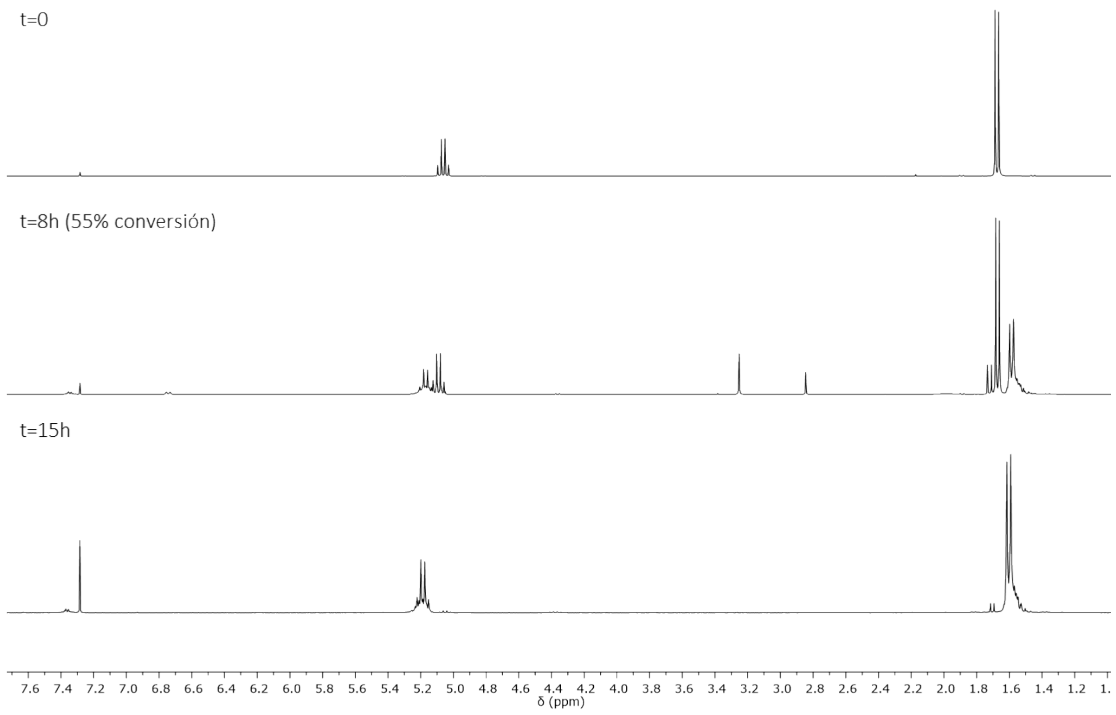

Figure S3. *1H NMR spectra of the polymerization kinetics of entry 4.*

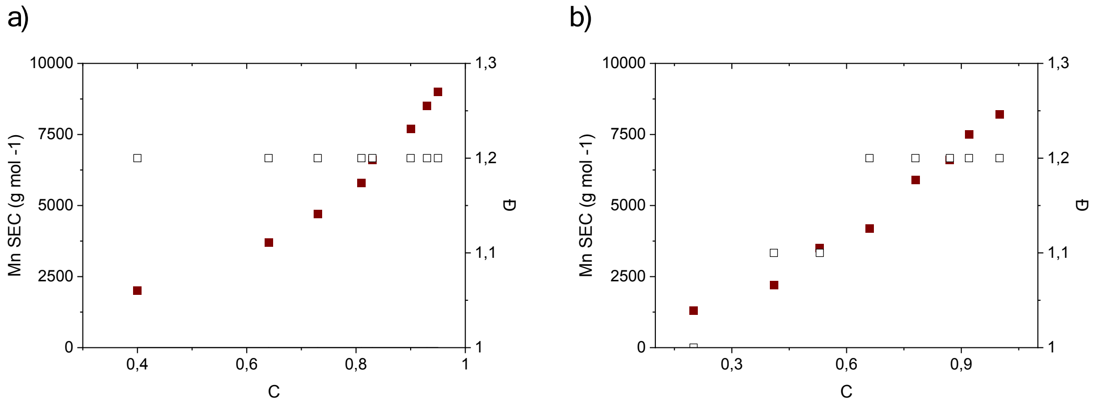

Figure S4. *Mn evolution of the polymerization employing a) DMAP:MSA (1:1) and b) DMAP:MSA (1:2), entries 5 & 6 respectively.*

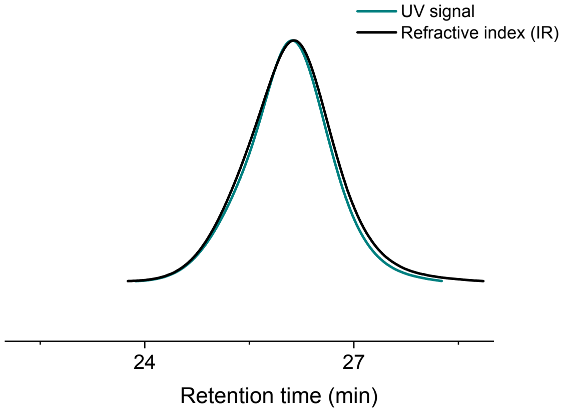

Figure S5. *UV-vis and RI SEC traces for PLLA.*

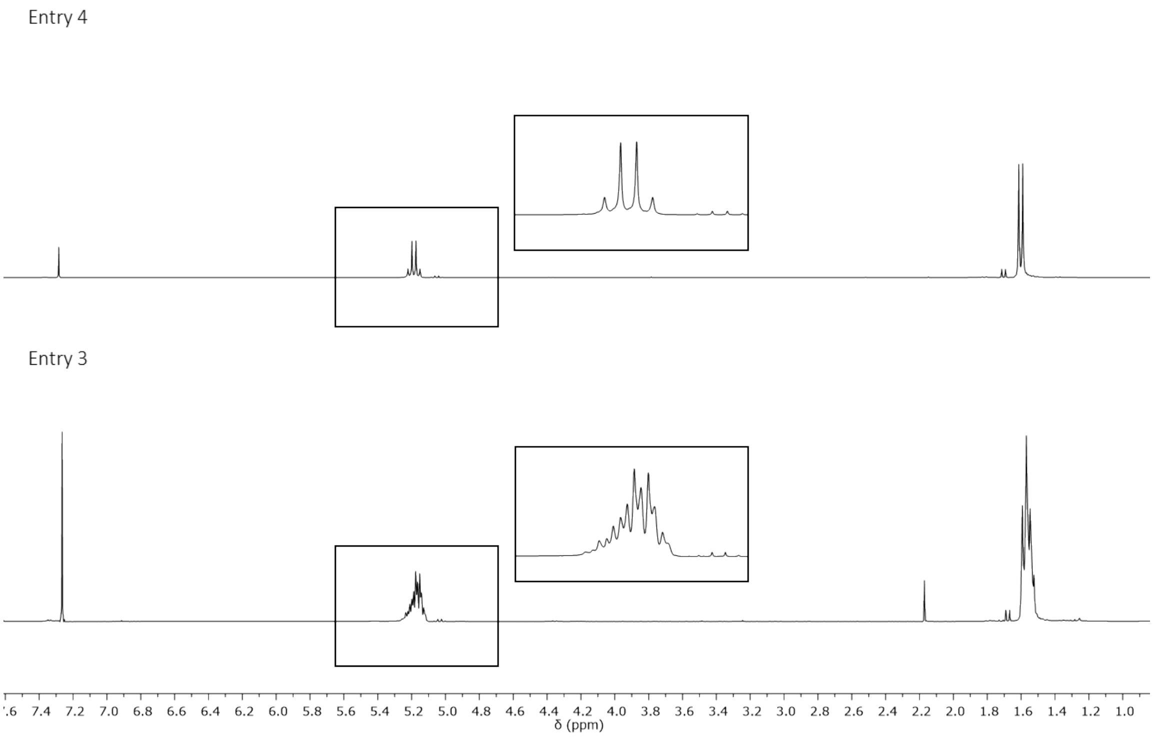

Figure S6. *^{1}H NMR of entry 4 and 3.*

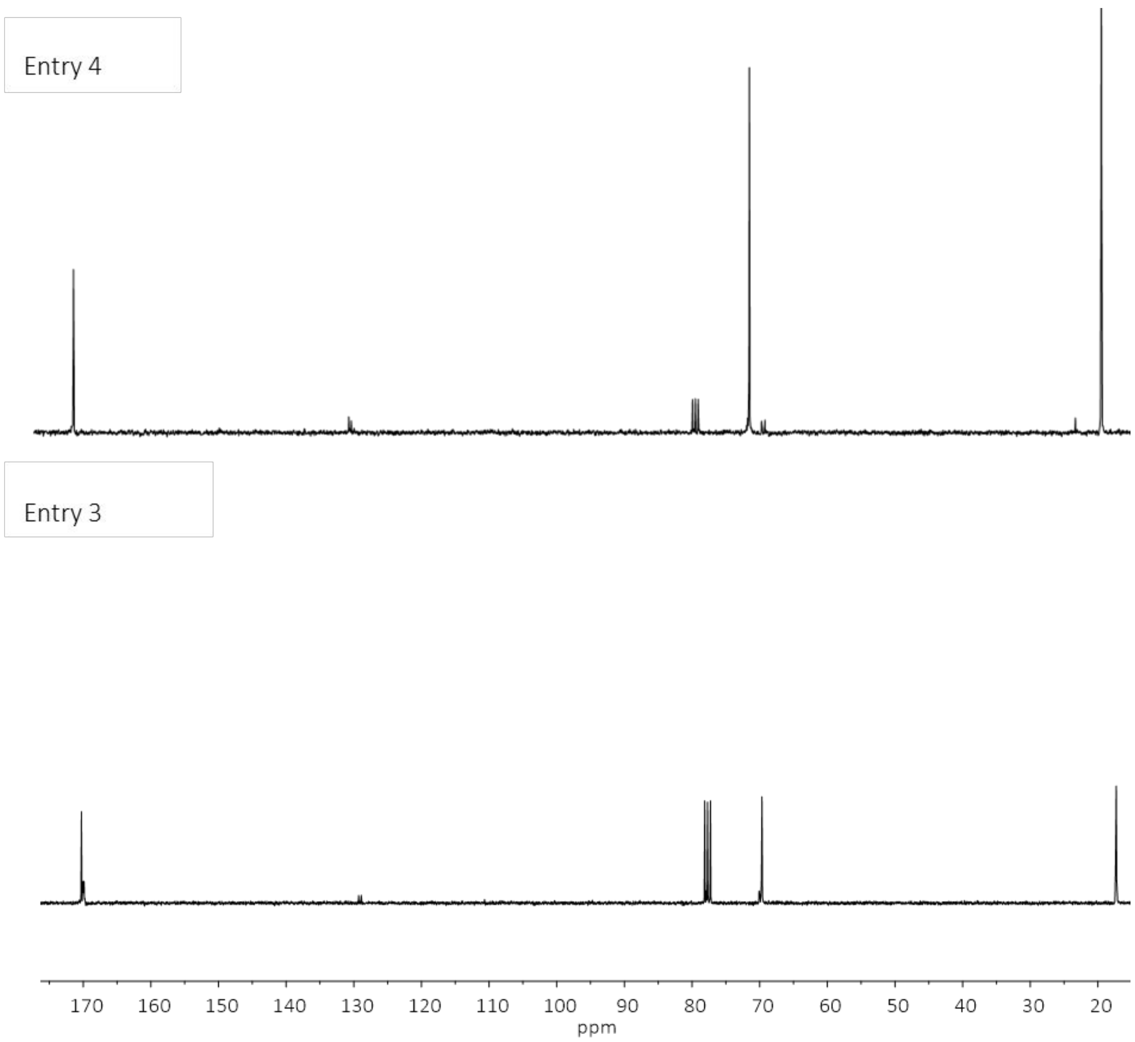

Figure S7. *^{13}C NMR of entry 4 and 3.*

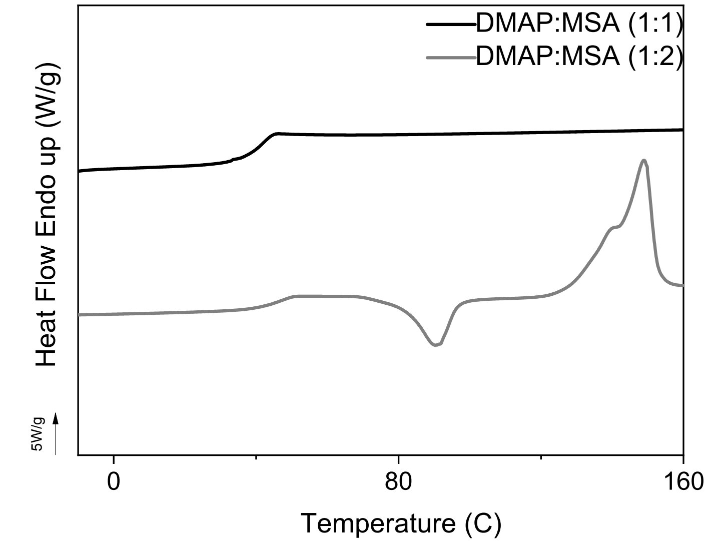

Figure S8. DSC analysis for PLA synthesized using DMAP:MSA (1:1) and DMAP:MSA (1:2).

The stereoregularity of PLA samples synthesized by DMAP:MSA (1:1) and DMAP:MSA (1:2) were determined by ^{13}C NMR taking into account the intensities of the methine region. P_m is defined as the probability of mesolinkage between all the monomer units and was calculated using the methine region of ^{13}C NMR spectrum: [mmm] = Pm(Pm+1)/2; [mrm] = (1-Pm)/2; [mmr] = Pm(1-Pm)/2; [rmm] = Pm (1-Pm)/2 and [rmr] = (1-Pm)2/2.

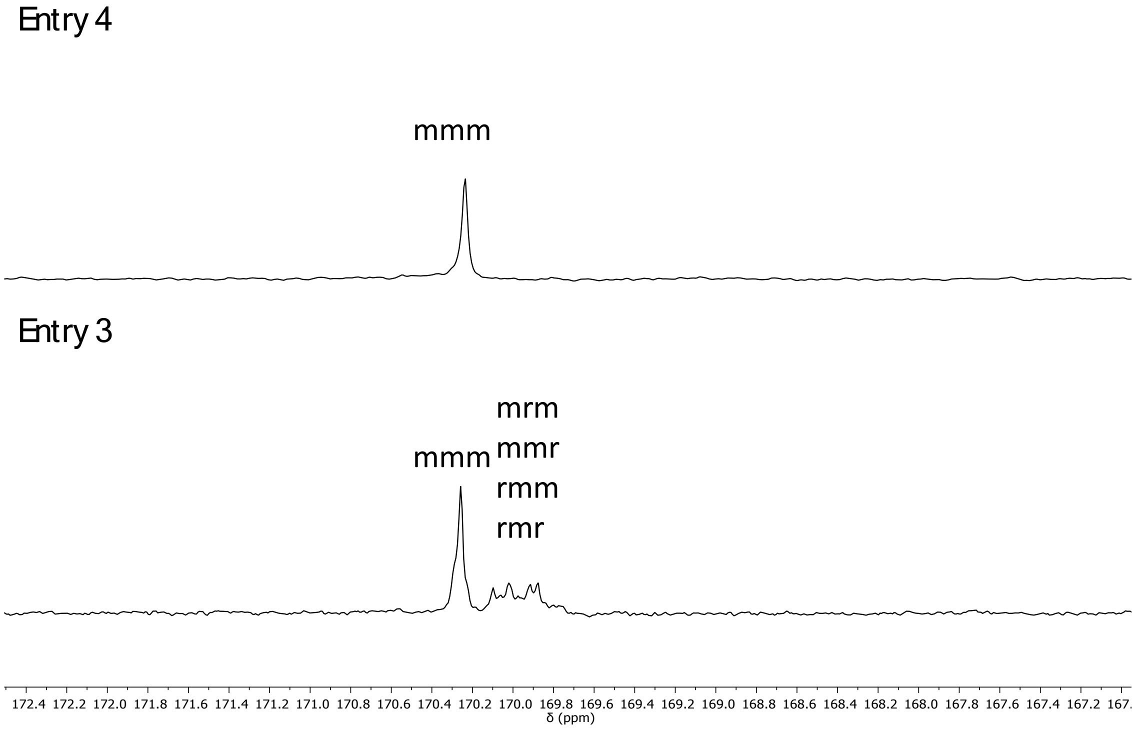

Figure S9. ^{13}C NMR analysis of PLA synthesized with DMAP:MSA (1:2) (Entry4) and DMAP:MSA (1:1) (Entry 3).

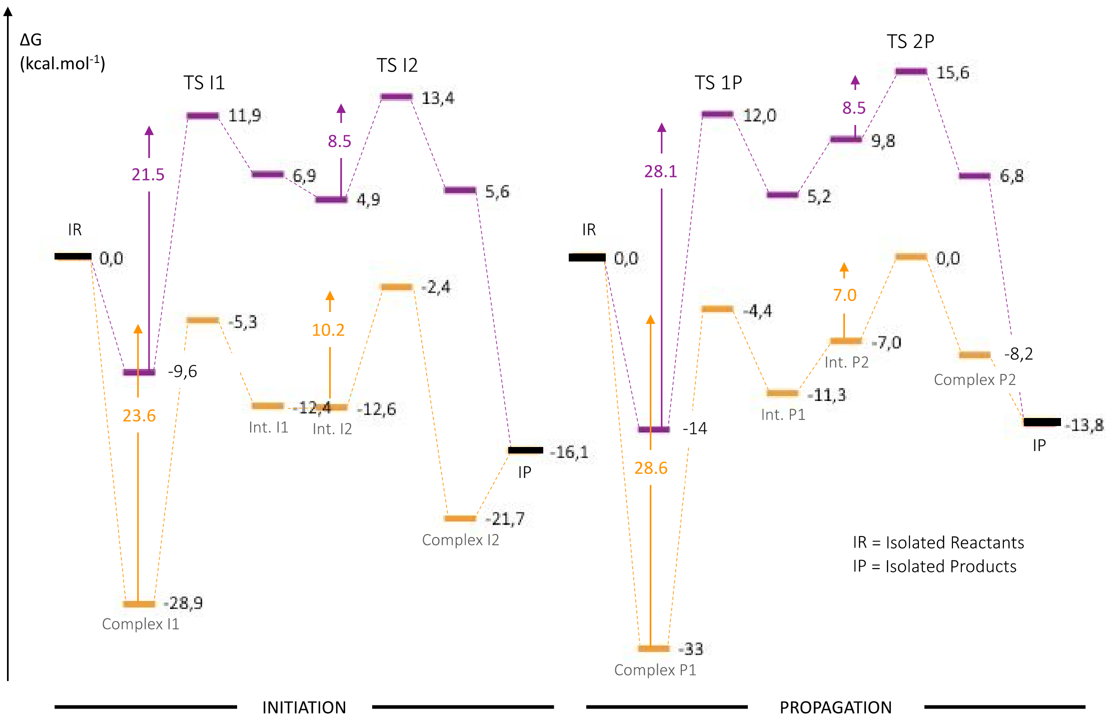

Figure S10. *Energy level diagram for the initiation and the propagation of the Ring-Opening Polymerization of L-lactide with energetic stationary levels calculated at the ωB97XD/6-311++G(2df,2p) level of theory catalyzed by DMAP:MSA (1:1) (in orange) and DMAP:MSA (1:2) (in purple).*

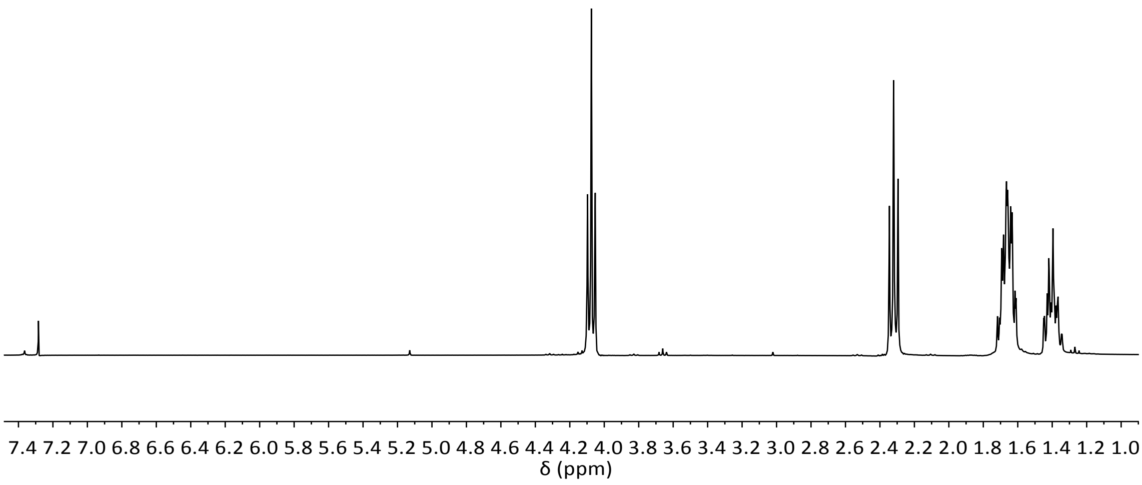

Figure S11. *^{1}H NMR spectrum of PCL catalyzed by DMAP:MSA (1:2).*

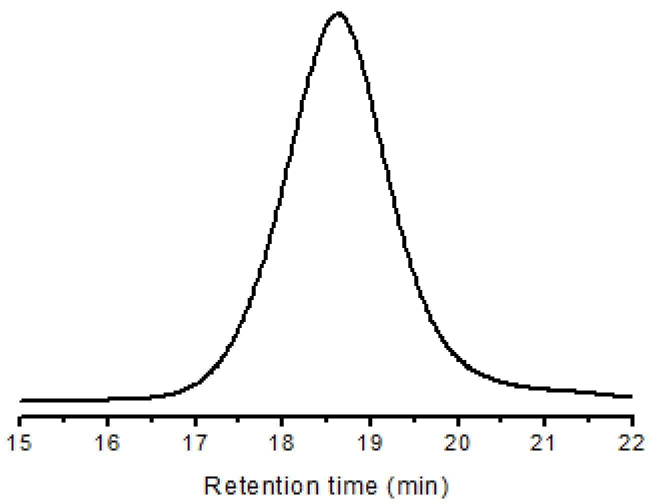

Figure S12. *SEC analysis of PCL catalyzed by DMAP:MSA (1:2).*

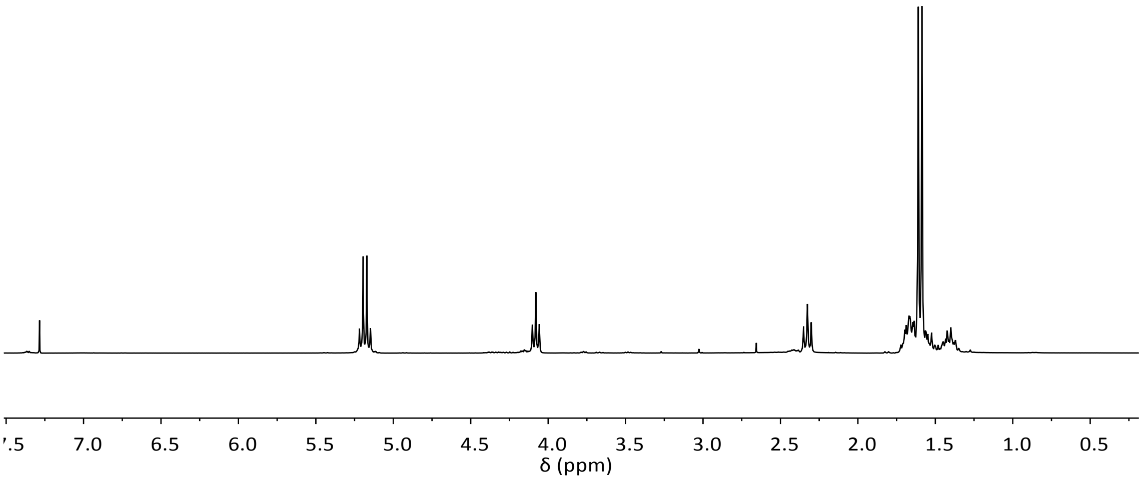

Figure S13. *^{1}H NMR spectra of PLLA-b-PCL copolymer.*

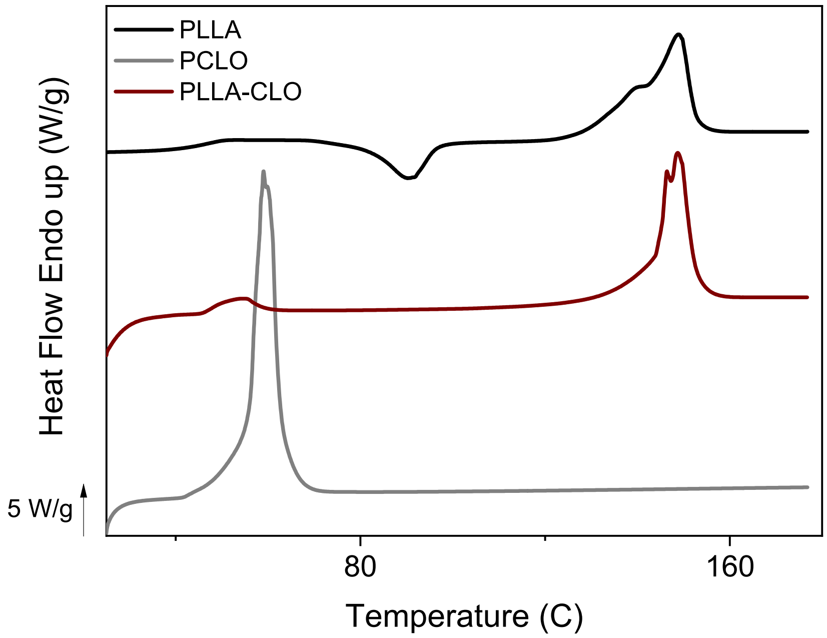

Figure S14. *DSC analysis of PLLA-b-PCL copolymer.*

Computational details

All calculations were carried out employing the Gaussian 16 suite of programs. Geometry optimizations were performed with the ωB97XD density functional and the 6-31+G(d,p) basis set. Frequency optimizations at the same level of theory confirmed that the optimized structures were minima (zero imaginary frequencies) or transition states (one imaginary frequency) and then used to evaluate the ZPVE and the thermal vibrational corrections at T = 298 K. The electronic energy was refined by single-point energy calculations at the ωB97XD/6-311++G(2df,2p) level of theory. The IRC method has been used to verify that the obtained transition states were connected to the desired minima.

Optimized cartesian coordinates

For DMAP:MSA (1:1) – Initiation

Complex I1

C	-4.084536	-1.206064	-1.072434
O	-1.976227	-0.105575	-1.856524
C	-1.956030	-2.297430	-0.764916
C	-3.411088	-0.167581	-1.952887
O	-5.266178	-1.129130	-0.850289
H	-1.580233	-2.564647	0.223298
C	-1.307898	-0.983645	-1.140964
O	-0.121346	-0.784503	-0.923164
C	-3.792699	-0.369258	-3.413079
H	-4.877681	-0.307911	-3.516988
H	-3.328837	0.409675	-4.022008
H	-3.457183	-1.345341	-3.775324
H	-3.758828	0.801431	-1.592065
C	3.117248	3.041617	-0.864111
C	4.226045	2.286994	-1.148288
N	2.112959	2.560613	-0.114030
C	4.320557	0.958552	-0.646871
H	5.004831	2.723150	-1.758151
C	2.166269	1.312493	0.388364
H	1.255788	3.163954	0.068641
C	3.241055	0.495253	0.150415
N	5.385288	0.172700	-0.915871
H	1.301548	1.018377	0.972693

H	3.231648	-0.502949	0.568164
C	6.491027	0.684771	-1.707868
C	5.443984	-1.184481	-0.382673
H	6.954502	1.557427	-1.232684
H	7.250243	-0.089947	-1.802037
H	6.164668	0.963248	-2.716654
H	4.576276	-1.773998	-0.690266
H	6.343298	-1.671858	-0.756207
H	5.478680	-1.182051	0.712075
O	-0.001890	4.081203	0.253130
S	-1.153150	3.340293	0.866702
O	-1.818978	4.048079	1.957552
O	-0.727558	1.943148	1.197216
C	-2.350218	3.167651	-0.454175
H	-2.643380	4.166477	-0.778622
H	-1.885744	2.609317	-1.268422
H	-3.210247	2.626703	-0.055353
H	-1.874226	0.694547	1.082127
O	0.903573	-1.539026	1.306638
H	2.995348	4.054923	-1.227750
H	0.508874	-1.303235	0.404168
O	-2.526069	-0.025449	0.929545
C	-3.356990	-0.149812	2.073642
H	-2.737360	-0.303588	2.967983
H	-3.956277	-1.053211	1.920140
C	-4.259052	1.061537	2.257643
H	-4.903286	0.929865	3.133454
H	-3.667504	1.971611	2.404904
H	-4.901921	1.189266	1.380412
S	1.579209	-2.972305	1.292782
O	3.002940	-2.833348	1.008764
O	-3.378487	-2.251404	-0.639465
C	-1.569463	-3.369284	-1.780035
H	-0.487901	-3.514344	-1.768363
H	-2.049548	-4.307641	-1.496328
H	-1.896867	-3.095322	-2.787608
C	1.379875	-3.395815	3.006015
H	0.314540	-3.443812	3.228786
H	1.852072	-4.368378	3.148278
H	1.874067	-2.634808	3.609461

O	0.787400	-3.872861	0.465489

TS I1

C	-4.610358	-0.943327	-0.659769
O	-2.534477	-0.040874	-1.758848
C	-2.480109	-2.021764	-0.381273
C	-3.968317	0.014791	-1.661475
O	-5.787991	-0.855695	-0.424728
H	-1.981662	-2.378509	0.520822
C	-1.877086	-0.696865	-0.809177
O	-0.599970	-0.613761	-0.952627
C	-4.538760	-0.184882	-3.057159
H	-5.621306	-0.050766	-3.028764
H	-4.100949	0.549985	-3.735812
H	-4.314129	-1.186419	-3.433062
H	-4.212069	1.014833	-1.292347
C	3.787460	2.666331	-0.593883
C	4.757668	1.743412	-0.883717
N	2.589370	2.295613	-0.110661
C	4.500537	0.358260	-0.671099
H	5.703198	2.094801	-1.272670
C	2.307362	0.994756	0.110381
H	1.863906	3.024352	0.093768
C	3.224026	0.012145	-0.150858
N	5.421826	-0.585166	-0.949836
H	1.316655	0.785906	0.493612
H	2.943859	-1.016148	0.049755
C	6.719908	-0.206446	-1.482207
C	5.110863	-1.996688	-0.730589
H	7.260624	0.448753	-0.789292
H	7.318165	-1.103601	-1.632149
H	6.623951	0.302733	-2.448543
H	4.245778	-2.311600	-1.320829
H	5.971691	-2.595648	-1.023655
H	4.881678	-2.198003	0.319321
O	0.760740	4.212050	0.387667
S	-0.603092	3.662069	0.627548
O	-1.184246	3.969861	1.933494

O	-0.594370	2.190460	0.305427
C	-1.650517	4.409984	-0.611738
H	-1.637435	5.488981	-0.455580
H	-1.258926	4.155479	-1.596733
H	-2.661220	4.020907	-0.482114
H	-1.680849	1.161619	0.484309
O	0.602796	-1.553060	0.987355
H	3.934402	3.729761	-0.738695
H	-0.081244	-1.093822	-0.184503
O	-2.313672	0.369577	0.609850
C	-2.183897	-0.085220	1.979551
H	-1.139759	-0.361148	2.155385
H	-2.812118	-0.974137	2.062605
C	-2.664102	0.999653	2.923007
H	-2.571630	0.642165	3.953320
H	-2.074142	1.914437	2.819982
H	-3.715405	1.234124	2.734452
S	1.073334	-2.998285	1.047681
O	2.452140	-3.125911	0.549205
O	-3.862302	-1.863657	-0.039570
C	-2.309251	-3.058269	-1.483139
H	-1.249702	-3.284783	-1.609995
H	-2.815958	-3.973759	-1.173119
H	-2.734044	-2.711291	-2.429216
C	1.120473	-3.342145	2.799326
H	0.114582	-3.225476	3.203395
H	1.465587	-4.368808	2.925306
H	1.811262	-2.643322	3.271300
O	0.077545	-3.897441	0.448084

Int. Ia

C	-4.817406	-0.815263	-0.493151
O	-2.832472	0.475176	-1.276628
C	-2.617604	-1.813672	-0.645007
C	-4.229446	0.506446	-0.994333
O	-5.995817	-0.898782	-0.247260
H	-2.110252	-2.491321	0.044232
C	-2.099865	-0.382425	-0.462250

O	-0.803885	-0.231784	-0.847646
C	-4.937977	0.974454	-2.255466
H	-6.009501	1.055515	-2.067241
H	-4.549086	1.952743	-2.547833
H	-4.769729	0.270834	-3.075230
H	-4.407647	1.218792	-0.179619
C	3.893388	2.731126	-0.017480
C	4.885994	1.885944	-0.432037
N	2.712038	2.258190	0.426863
C	4.671096	0.475049	-0.409985
H	5.814990	2.315931	-0.779823
C	2.471281	0.926384	0.474790
H	1.965739	2.921866	0.681124
C	3.413156	0.021702	0.074977
N	5.607133	-0.392704	-0.830178
H	1.499014	0.612256	0.833599
H	3.156058	-1.032711	0.125052
C	6.897496	0.088389	-1.297838
C	5.339762	-1.832005	-0.793226
H	7.423286	0.646812	-0.514784
H	7.514772	-0.763952	-1.575766
H	6.787987	0.729883	-2.179761
H	4.432371	-2.084474	-1.346997
H	6.182778	-2.355857	-1.240623
H	5.206095	-2.184627	0.233681
O	0.578758	4.066939	0.699906
S	-0.690355	3.374602	0.436845
O	-1.934080	4.009374	0.819441
O	-0.505452	1.982037	1.169918
C	-0.747356	2.954497	-1.288478
H	-0.810782	3.896967	-1.834295
H	0.159907	2.405945	-1.540835
H	-1.619238	2.323203	-1.468276
H	-1.267270	1.332856	1.028672
O	0.723038	-1.564258	0.815933
H	4.003537	3.808493	-0.028864
H	-0.216903	-0.810139	-0.288790
O	-2.305000	0.114233	0.875950
C	-2.183632	-0.777904	2.007824
H	-1.195901	-1.248572	1.983904

H	-2.963498	-1.540866	1.931933
C	-2.378890	0.051462	3.260188
H	-2.341235	-0.603685	4.135149
H	-1.596703	0.807739	3.366308
H	-3.350661	0.552187	3.245969
S	1.128256	-2.980810	0.483156
O	2.546201	-3.046495	0.072432
O	-4.012709	-1.863746	-0.276122
C	-2.423680	-2.323183	-2.060018
H	-1.358150	-2.454004	-2.252953
H	-2.913335	-3.294801	-2.152738
H	-2.847783	-1.630045	-2.791608
C	1.021978	-3.846851	2.045437
H	-0.010423	-3.805361	2.395121
H	1.322569	-4.881265	1.875931
H	1.691098	-3.364142	2.758221
O	0.164856	-3.618966	-0.425095

Int. Ib

C	4.085744	-1.789972	0.109306
O	2.114241	-0.312371	-0.163629
C	2.359750	-1.624759	1.805386
C	3.323428	-0.783828	-0.756269
O	5.132627	-2.246708	-0.277993
H	1.914569	-2.433547	2.388274
C	1.426562	-1.270870	0.638864
O	0.305112	-0.695472	1.126732
C	4.190509	0.423972	-1.075241
H	5.102965	0.097403	-1.575817
H	3.638759	1.103086	-1.730272
H	4.467040	0.949743	-0.154719
H	3.071062	-1.320395	-1.676658
C	-1.619048	1.711334	1.679630
C	-2.525706	0.711312	1.876261
N	-1.813598	2.657184	0.739052
C	-3.669919	0.614114	1.033536
H	-2.287448	-0.054161	2.599584
C	-2.917516	2.639560	-0.038702

H	-1.057907	3.312447	0.521335
C	-3.866609	1.672883	0.099672
N	-4.500572	-0.437533	1.088299
H	-2.980996	3.410606	-0.795220
H	-4.695711	1.666245	-0.591106
C	-4.137442	-1.601504	1.889498
C	-5.549376	-0.587284	0.081392
H	-4.024897	-1.328921	2.943298
H	-4.937355	-2.336803	1.819828
H	-3.214769	-2.048738	1.501775
H	-5.107885	-0.636196	-0.920511
H	-6.091238	-1.510618	0.278856
H	-6.262648	0.240963	0.144018
O	0.447619	4.163651	-0.105225
S	1.575532	3.297946	-0.453934
O	1.697196	2.778991	-1.801193
O	1.562655	2.118754	0.603623
C	3.063605	4.169959	-0.021828
H	3.116739	5.051592	-0.661508
H	3.006948	4.456856	1.027805
H	3.911257	3.509788	-0.206777
H	1.743367	1.212677	0.203087
O	-1.125717	0.094344	-0.865108
H	-0.684698	1.763187	2.222925
H	-0.295949	-0.446864	0.351889
O	1.202429	-2.344232	-0.223827
C	0.548480	-3.487477	0.338524
H	-0.342302	-3.156766	0.881886
H	1.238180	-3.996742	1.024801
C	0.162399	-4.412147	-0.795288
H	-0.237683	-5.343779	-0.383981
H	-0.613684	-3.950999	-1.408323
H	1.032865	-4.653288	-1.412429
S	-1.980901	-0.926233	-1.563793
O	-3.187936	-0.326434	-2.162919
O	3.583349	-2.179518	1.289807
C	2.667943	-0.449058	2.714723
H	1.746089	-0.079475	3.167739
H	3.355074	-0.767488	3.501972
H	3.129980	0.369092	2.155383

C	-0.962937	-1.502108	-2.916313
H	-0.032912	-1.886002	-2.494202
H	-1.505697	-2.283409	-3.450105
H	-0.767096	-0.653794	-3.572732
O	-2.247593	-2.094185	-0.693622

TS2 I2

C	-3.835974	1.984178	0.181494
O	-1.995541	0.402912	-0.322027
C	-2.118497	1.584152	1.875177
C	-3.265570	0.906509	-0.743948
O	-4.806656	2.606129	-0.172684
H	-1.688801	2.313981	2.569242
C	-1.079249	1.381869	0.772291
O	-0.048673	0.730558	1.073313
C	-4.272191	-0.223647	-0.923593
H	-5.201548	0.192652	-1.315188
H	-3.873245	-0.962053	-1.623252
H	-4.480423	-0.715187	0.031777
H	-3.098551	1.410243	-1.699284
C	1.313225	-1.997835	1.401189
C	2.338575	-1.159618	1.740912
N	1.426212	-2.859688	0.374076
C	3.525251	-1.139862	0.959624
H	2.167877	-0.445785	2.532978
C	2.560362	-2.921738	-0.349437
H	0.582779	-3.406730	0.089427
C	3.624021	-2.108379	-0.078889
N	4.490604	-0.224017	1.165029
H	2.563708	-3.628477	-1.169625
H	4.489037	-2.158951	-0.723568
C	4.258642	0.874955	2.095158
C	5.637297	-0.157705	0.265143
H	4.100142	0.501153	3.111910
H	5.138579	1.515850	2.107309
H	3.399708	1.477745	1.778625
H	5.315376	0.071001	-0.756719
H	6.305077	0.631546	0.606737

H	6.196402	-1.098929	0.281666
O	-0.855936	-4.090302	-0.329244
S	-2.000123	-3.159039	-0.524837
O	-2.228762	-2.725303	-1.904479
O	-1.900220	-2.001679	0.440898
C	-3.440822	-4.071500	0.000938
H	-3.535268	-4.946466	-0.642613
H	-3.305383	-4.369982	1.040415
H	-4.311068	-3.422983	-0.107239
H	-1.978704	-0.589383	-0.045291
O	1.384602	0.142098	-0.861342
H	0.346973	-1.973271	1.889536
H	0.796449	0.463935	-0.049124
O	-0.967811	2.454158	-0.104202
C	-0.432461	3.644108	0.498973
H	0.480396	3.384493	1.045617
H	-1.176680	4.058852	1.190685
C	-0.130600	4.637357	-0.601142
H	0.169951	5.590802	-0.157247
H	0.691661	4.281241	-1.224627
H	-1.015746	4.809360	-1.219940
S	2.258432	1.275664	-1.490083
O	3.428418	0.649669	-2.094434
O	-3.294628	2.241517	1.381796
C	-2.471029	0.326372	2.646922
H	-1.564439	-0.098542	3.082262
H	-3.169732	0.580270	3.447211
H	-2.921822	-0.434287	2.006213
C	1.206017	1.887494	-2.788274
H	0.268695	2.208708	-2.331372
H	1.718471	2.719080	-3.273665
H	1.036917	1.072668	-3.492171
O	2.472776	2.341131	-0.511458

Complex I2

C	4.294554	-1.661501	0.715407
O	2.657888	-0.380535	-0.543882
C	2.009648	-1.754557	1.742119

C	4.025667	-0.523580	-0.271669
O	5.403868	-2.112927	0.849713
H	1.625422	-2.535584	2.412883
C	0.967180	-1.693397	0.623143
O	0.046839	-0.893138	0.730748
C	4.722555	0.757514	0.186284
H	5.784122	0.555539	0.343163
H	4.604753	1.519081	-0.588389
H	4.288752	1.136016	1.116007
H	4.526697	-0.864963	-1.188311
C	-1.259766	1.887247	0.865699
C	-2.332554	1.268762	1.449665
N	-1.388274	2.561761	-0.291329
C	-3.605487	1.304741	0.818933
H	-2.163470	0.696937	2.350306
C	-2.576912	2.630999	-0.917598
H	-0.542296	3.035275	-0.699262
C	-3.691552	2.029189	-0.403297
N	-4.674207	0.670872	1.338390
H	-2.590716	3.177426	-1.852360
H	-4.612464	2.080084	-0.965459
C	-4.532376	-0.121335	2.552650
C	-5.916915	0.595990	0.580042
H	-4.153904	0.494856	3.374885
H	-5.511928	-0.498476	2.842770
H	-3.864964	-0.972977	2.383138
H	-5.749537	0.110208	-0.387149
H	-6.636726	0.004236	1.143234
H	-6.344715	1.593301	0.428707
O	0.762535	3.850152	-1.209233
S	2.052338	3.229430	-0.782281
O	2.966800	2.891603	-1.874761
O	1.790713	2.082709	0.151316
C	2.864697	4.471306	0.214564
H	3.048673	5.341814	-0.415618
H	2.215320	4.732228	1.050567
H	3.806839	4.054023	0.572519
H	2.360459	0.546580	-0.396768
O	-1.742162	-0.655502	-1.119216
H	-0.250777	1.838039	1.258879

H	-1.034026	-0.785543	-0.405213
O	0.867921	-2.615449	-0.321945
C	1.921710	-3.521193	-0.714443
H	2.305657	-4.048548	0.160112
H	2.722804	-2.928596	-1.160748
C	1.322828	-4.474168	-1.724593
H	2.096693	-5.163288	-2.073549
H	0.516430	-5.063898	-1.279078
H	0.932984	-3.929224	-2.588409
S	-2.918228	-1.696146	-0.956563
O	-4.079956	-1.132586	-1.626039
O	3.289531	-2.277910	1.394701
C	2.086954	-0.452895	2.525932
H	1.140788	-0.278186	3.041149
H	2.880890	-0.541609	3.271538
H	2.276381	0.403003	1.876331
C	-2.330665	-3.089159	-1.894136
H	-1.388740	-3.413411	-1.450682
H	-3.086911	-3.872205	-1.829887
H	-2.187480	-2.769847	-2.926268
O	-3.031578	-2.098549	0.441201

For DMAP:MSA (1:1) – Propagation

Complex P1

C	-1.590688	-2.809564	-0.408577
O	0.377192	-2.396415	-1.748032
C	0.484553	-2.341103	0.660241
C	-0.973664	-1.941418	-1.495243
O	-2.702985	-3.273659	-0.439387
H	0.277139	-1.272592	0.800208
C	1.177667	-2.532556	-0.684520
O	2.345449	-2.806781	-0.825405
C	-1.741208	-1.974140	-2.793062
H	-2.745266	-1.580819	-2.621777
H	-1.234452	-1.349874	-3.532437
H	-1.824534	-2.992917	-3.177631
H	-0.912484	-0.918801	-1.107280
C	4.243654	2.279404	-0.995098
C	5.206795	1.397298	-0.593446
N	3.236281	1.895697	-1.802847
C	5.112522	0.027860	-0.968589
H	5.962975	1.745560	0.094342
C	3.158116	0.622574	-2.245872
H	2.404846	2.505355	-1.912464
C	4.077208	-0.317407	-1.882895
N	5.940002	-0.900860	-0.462227
H	2.298878	0.382351	-2.857205
H	3.925312	-1.334432	-2.214563
C	6.909110	-0.530099	0.562610
C	5.738270	-2.310093	-0.785583
H	6.401528	-0.104311	1.434479
H	7.451655	-1.421524	0.873154
H	7.638190	0.187697	0.170963
H	4.728737	-2.636266	-0.516606
H	5.900299	-2.493108	-1.853407
H	6.460786	-2.904743	-0.228998
O	0.785820	3.100328	-1.718388
S	-0.135855	2.123726	-1.085623
O	-0.831949	2.612365	0.119922
O	0.554220	0.809250	-0.832688

C	-1.378762	1.762896	-2.310254
H	-1.864339	2.704293	-2.569627
H	-0.885185	1.337394	-3.184639
H	-2.094364	1.066704	-1.872580
H	-0.984574	1.215107	1.269809
O	2.362183	0.117689	0.854862
H	4.213838	3.303140	-0.646733
H	1.649002	0.553634	0.283889
O	-1.032002	0.466106	1.900842
C	-2.370350	0.254508	2.257132
H	-2.380607	-0.532359	3.017743
S	2.906600	1.011446	2.047222
O	2.772714	2.418432	1.699491
O	-0.782617	-3.037687	0.638105
C	1.287673	-2.877830	1.820084
H	2.249429	-2.363591	1.854033
H	0.743369	-2.697976	2.749938
H	1.463871	-3.950054	1.708743
C	1.796205	0.626764	3.379738
H	1.929371	-0.426105	3.629286
H	2.079606	1.260706	4.220932
H	0.771420	0.820332	3.053936
O	4.221719	0.475906	2.373642
C	-5.169839	-1.236201	0.351860
H	-4.618812	-1.876102	-0.341448
C	-3.037373	1.520020	2.805559
H	-3.016734	2.310081	2.048722
H	-4.075444	1.325565	3.085953
H	-2.483925	1.864730	3.682094
C	-6.338071	-1.985838	0.968653
H	-5.968393	-2.876812	1.480181
H	-6.870417	-1.355552	1.685983
H	-7.027920	-2.293670	0.179656
C	-5.872322	2.340389	-0.402029
H	-6.741036	2.194884	-1.048588
H	-6.127163	3.034264	0.400531
C	-4.662419	2.812278	-1.186613
H	-4.449684	2.123990	-2.007953
H	-4.862435	3.801995	-1.608448
H	-3.779639	2.878610	-0.544063

C	-3.167567	-0.245743	1.061565
C	-5.640159	-0.018586	-0.436310
O	-4.295257	-0.855811	1.425375
O	-5.585159	1.105104	0.281505
O	-6.052680	-0.099616	-1.570355
O	-2.844755	-0.072945	-0.095030

TS P1

C	-0.053885	-4.249565	-0.664764
O	-1.226914	-2.195076	0.057653
C	-0.763396	-2.543198	-2.245890
C	-0.597928	-3.403312	0.487430
O	0.459725	-5.318182	-0.448991
H	-0.216780	-2.160605	-3.110971
C	-0.605929	-1.571346	-1.069821
O	-1.263084	-0.425573	-1.339897
C	-1.601319	-4.192688	1.312218
H	-1.128652	-5.101998	1.685669
H	-1.936863	-3.584081	2.155176
H	-2.464290	-4.475915	0.699367
H	0.272977	-3.138759	1.096053
C	-3.682870	1.547232	-1.017382
C	-2.931729	2.461380	-1.696894
N	-4.136065	1.799827	0.226598
C	-2.565352	3.687715	-1.071757
H	-2.542023	2.181088	-2.664067
C	-3.839161	2.962193	0.846354
H	-4.625691	1.065259	0.745034
C	-3.091097	3.920214	0.232876
N	-1.746087	4.569147	-1.663166
H	-4.202023	3.069227	1.860107
H	-2.829038	4.803655	0.794451
C	-1.097979	4.220619	-2.921506
C	-1.202123	5.682015	-0.887548
H	-1.843707	3.987942	-3.687810
H	-0.523024	5.078008	-3.268173
H	-0.418962	3.373931	-2.770465
H	-0.678170	5.305225	-0.001788

H	-0.496399	6.228075	-1.511097
H	-1.998678	6.373655	-0.593351
O	-5.272965	-0.342798	1.712462
S	-4.392754	-1.512228	1.732170
O	-3.380175	-1.631397	2.761622
O	-3.739719	-1.589760	0.290279
C	-5.428786	-2.956508	1.749613
H	-5.980973	-2.943991	2.689950
H	-6.110037	-2.906765	0.900698
H	-4.787597	-3.836350	1.692605
H	-2.750899	-1.789630	0.300093
O	-1.030810	1.234462	0.622613
H	-3.907284	0.566637	-1.416142
H	-1.109236	0.244017	-0.593941
O	0.717412	-1.398694	-0.655314
C	1.759414	-1.251091	-1.603549
H	1.803844	-2.135009	-2.256725
S	0.058739	2.270951	0.609588
O	-0.294487	3.463479	1.403454
O	-0.117348	-3.788967	-1.927461
C	-2.209983	-2.789868	-2.632643
H	-2.672811	-1.853611	-2.950147
H	-2.247864	-3.508437	-3.454403
H	-2.778756	-3.189500	-1.788982
C	1.446641	1.498557	1.429626
H	1.639287	0.531753	0.963236
H	2.309180	2.157553	1.317921
H	1.181948	1.362766	2.479400
O	0.499443	2.574446	-0.771322
C	1.664218	0.024716	-2.436562
H	1.596597	0.907234	-1.795146
H	2.541148	0.103957	-3.082138
H	0.765461	-0.000712	-3.057352
C	5.361672	-1.073777	-0.847938
H	5.496485	-1.974780	-0.244136
C	6.457468	-0.933421	-1.890993
H	6.446289	-1.797687	-2.558681
H	6.306361	-0.025690	-2.480416
H	7.431867	-0.882690	-1.398854
C	6.098825	0.949776	2.159961

H	6.066599	1.937337	1.695493
H	7.030470	0.827383	2.713134
C	4.894982	0.744371	3.071027
H	4.930396	-0.260685	3.507572
H	3.976558	0.825968	2.481246
O	4.118347	-1.153013	-1.547497
O	6.114632	-0.071330	1.147203
O	4.958338	1.722708	4.095013
H	4.106454	2.154824	4.179884
C	3.032710	-1.291679	-0.769346
C	5.327883	0.133625	0.087270
O	3.086658	-1.434388	0.427700
O	4.700131	1.141992	-0.127909

Int. P1

C	-0.591438	-3.804783	0.298468
O	-1.335885	-1.641013	1.289603
C	-1.267384	-1.930283	-1.069572
C	-0.892926	-2.966678	1.543544
O	-0.198003	-4.940366	0.412209
H	-0.855729	-1.551621	-2.006371
C	-0.824880	-1.070610	0.122800
O	-1.292098	0.192805	0.112810
C	-1.948345	-3.642132	2.406387
H	-1.618108	-4.644539	2.683063
H	-2.105199	-3.050562	3.311762
H	-2.896507	-3.719396	1.865604
H	0.058349	-2.926598	2.088448
C	-2.549132	1.325896	2.730276
C	-3.582539	0.535123	2.321472
N	-2.143668	2.378305	1.993017
C	-4.209776	0.781145	1.067758
H	-3.851934	-0.310885	2.936296
C	-2.732060	2.667304	0.811876
H	-1.296617	2.861339	2.297264
C	-3.773715	1.922932	0.340005
N	-5.153167	-0.044876	0.581805
H	-2.304265	3.481670	0.242639

H	-4.151458	2.143701	-0.649879
C	-5.540052	-1.227385	1.333329
C	-5.819377	0.251405	-0.686137
H	-6.037398	-0.965562	2.275429
H	-6.231624	-1.817895	0.734701
H	-4.666018	-1.849867	1.554329
H	-6.450586	1.143241	-0.594238
H	-5.094474	0.410060	-1.490485
H	-6.453401	-0.592918	-0.952796
O	0.439273	2.747173	3.163286
S	1.305535	1.906115	2.331485
O	1.439634	2.253738	0.926813
O	0.762796	0.433750	2.500930
C	2.911220	1.824808	3.081270
H	3.352221	2.820057	3.015070
H	2.788286	1.520461	4.120357
H	3.492685	1.093409	2.518426
H	0.864131	-0.105356	1.663429
O	-1.441141	1.754154	-1.958053
H	-1.987880	1.140315	3.636617
H	-1.254811	0.676272	-0.760521
O	0.606212	-1.098864	0.321045
C	1.512958	-1.088381	-0.778741
H	1.386641	-2.007320	-1.361277
S	-2.238149	1.251239	-3.127155
O	-3.694949	1.301982	-2.866589
O	-0.719902	-3.258522	-0.917254
C	-2.777575	-1.999130	-1.200669
H	-3.180097	-1.005313	-1.401186
H	-3.030660	-2.641741	-2.045632
H	-3.230929	-2.395925	-0.287779
C	-1.922889	2.428042	-4.435038
H	-0.852107	2.433270	-4.639971
H	-2.481171	2.107533	-5.315080
H	-2.257937	3.411509	-4.104678
O	-1.742306	-0.056643	-3.598147
C	5.157107	-1.576243	-0.563959
H	5.255919	-1.965873	0.452259
C	1.418988	0.144777	-1.678219
H	1.451809	1.052877	-1.073630

H	2.262176	0.138471	-2.373888
H	0.497420	0.144261	-2.262365
C	5.963330	-2.407602	-1.549135
H	5.618183	-3.443313	-1.526193
H	5.847521	-2.019885	-2.564448
H	7.019117	-2.377698	-1.271708
C	5.265287	2.086125	-1.248896
H	6.350790	2.166041	-1.344351
H	4.788495	2.477968	-2.148316
C	4.755550	2.781132	-0.001623
H	5.264285	2.395111	0.885558
H	4.960684	3.853671	-0.074936
H	3.676869	2.639553	0.105979
C	2.888566	-1.119112	-0.139297
C	5.660299	-0.135943	-0.527871
O	3.786827	-1.653679	-0.970065
O	4.903018	0.688969	-1.245592
O	6.671200	0.173057	0.060555
O	3.158535	-0.678121	0.956110

Int. P2

C	0.212079	-4.177425	-0.698633
O	-1.071800	-2.190497	0.021903
C	-0.461313	-2.438210	-2.259864
C	-0.425489	-3.390557	0.447731
O	0.747209	-5.237013	-0.489830
H	0.121868	-2.007084	-3.076924
C	-0.407000	-1.505134	-1.043658
O	-1.086304	-0.372537	-1.312558
C	-1.445137	-4.240976	1.187474
H	-0.962493	-5.146232	1.558015
H	-1.849078	-3.673126	2.028891
H	-2.261617	-4.531766	0.517136
H	0.400011	-3.117965	1.113461
C	-3.591856	1.499182	-1.067731
C	-2.824024	2.451918	-1.671370
N	-4.125719	1.700730	0.153335
C	-2.526199	3.665108	-0.986939

H	-2.369334	2.211039	-2.620577
C	-3.897093	2.849566	0.825107
H	-4.622955	0.936734	0.619145
C	-3.138230	3.844244	0.288090
N	-1.691204	4.582041	-1.497027
H	-4.322263	2.915302	1.817921
H	-2.933007	4.714550	0.892050
C	-0.951463	4.284474	-2.717317
C	-1.222578	5.679548	-0.653078
H	-1.637707	4.058253	-3.539105
H	-0.374637	5.163827	-2.999968
H	-0.264264	3.448850	-2.543115
H	-0.750602	5.284723	0.253835
H	-0.488472	6.257882	-1.211330
H	-2.051075	6.346541	-0.391960
O	-5.271268	-0.530466	1.497660
S	-4.352062	-1.669254	1.530335
O	-3.400203	-1.793048	2.615442
O	-3.611517	-1.671894	0.129538
C	-5.335119	-3.147257	1.433609
H	-5.942924	-3.188113	2.338158
H	-5.965471	-3.089084	0.546752
H	-4.660613	-4.002355	1.385298
H	-2.618473	-1.839463	0.192148
O	-1.034899	1.205139	0.720874
H	-3.766715	0.526880	-1.509243
H	-1.002043	0.272589	-0.532562
O	0.882798	-1.306293	-0.544543
C	1.971477	-1.085943	-1.423555
H	2.081857	-1.939155	-2.108856
S	0.021233	2.271433	0.833648
O	-0.431066	3.418758	1.644068
O	0.206947	-3.673656	-1.946236
C	-1.873222	-2.717333	-2.741103
H	-2.348337	-1.785553	-3.053601
H	-1.838076	-3.406687	-3.587674
H	-2.476477	-3.165081	-1.947009
C	1.366897	1.503601	1.722683
H	1.630552	0.569522	1.225623
H	2.211000	2.194385	1.716689

H	1.028959	1.306776	2.740767
O	0.549791	2.648166	-0.498249
C	1.881286	0.220725	-2.207770
H	1.743001	1.071204	-1.535166
H	2.792832	0.357139	-2.793102
H	1.023749	0.191807	-2.884357
C	5.524781	-0.866804	-0.481748
H	5.625760	-1.764637	0.133557
C	6.671770	-0.742022	-1.471504
H	6.686016	-1.611234	-2.132746
H	6.556457	0.161568	-2.075470
H	7.621223	-0.694235	-0.932949
C	6.278057	1.219641	2.483712
H	6.232591	2.187601	1.979258
H	7.242508	1.112489	2.982629
O	4.318755	-0.943248	-1.240505
O	6.275321	0.175276	1.487616
C	3.197980	-1.124682	-0.521818
C	5.459888	0.346203	0.444968
O	3.192932	-1.305175	0.670617
O	4.795576	1.331809	0.233908
C	5.121486	1.040674	3.449011
H	4.165970	1.121266	2.925918
H	5.172292	0.064326	3.937566
H	5.161647	1.818432	4.217609

TS P2

C	0.156738	-4.031379	-0.739707
O	-1.078932	-2.077003	0.142512
C	-0.569378	-2.239443	-2.240654
C	-0.480411	-3.341618	0.466519
O	0.745837	-5.072155	-0.586625
H	-0.006637	-1.827859	-3.083983
C	-0.418770	-1.260472	-1.070940
O	-1.044344	-0.158584	-1.195277
C	-1.498700	-4.255096	1.132597
H	-0.994150	-5.173797	1.434545
H	-1.922918	-3.759593	2.008434

H	-2.306726	-4.509465	0.439862
H	0.343689	-3.123704	1.150306
C	-3.599067	1.395564	-0.902171
C	-2.922869	2.412479	-1.517005
N	-4.096580	1.537675	0.339678
C	-2.691895	3.632149	-0.824297
H	-2.488973	2.220458	-2.486941
C	-3.924452	2.685785	1.023191
H	-4.552886	0.720529	0.792786
C	-3.253154	3.742762	0.480045
N	-1.956653	4.623442	-1.359566
H	-4.326542	2.706725	2.028001
H	-3.097942	4.621160	1.088353
C	-1.285279	4.418232	-2.636560
C	-1.578505	5.770809	-0.541249
H	-2.010460	4.178213	-3.420661
H	-0.781606	5.341025	-2.920457
H	-0.537690	3.621856	-2.552323
H	-1.022210	5.445554	0.344686
H	-0.941479	6.426838	-1.132130
H	-2.463192	6.342061	-0.239755
O	-5.210762	-0.675061	1.437372
S	-4.341982	-1.877353	1.431535
O	-3.528235	-2.084093	2.627786
O	-3.517473	-1.907562	0.154548
C	-5.448989	-3.266001	1.270967
H	-6.104002	-3.269665	2.142538
H	-6.026747	-3.148207	0.354420
H	-4.849675	-4.176553	1.240332
H	-2.154335	-2.006180	0.183406
O	-0.891309	1.360879	0.721932
H	-3.721637	0.417317	-1.349121
H	-0.889898	0.704895	-0.138926
O	0.880779	-1.210979	-0.542325
C	1.981331	-1.092343	-1.427264
H	2.024329	-1.962818	-2.098066
S	0.236174	2.436109	0.726182
O	-0.197352	3.544165	1.573451
O	0.083538	-3.493004	-1.967538
C	-2.004799	-2.469178	-2.673278

H	-2.457012	-1.514662	-2.949037
H	-2.017713	-3.137947	-3.536840
H	-2.603884	-2.909879	-1.873286
C	1.593520	1.606842	1.523436
H	1.745059	0.640423	1.041741
H	2.479611	2.233614	1.415028
H	1.329470	1.468541	2.572067
O	0.629957	2.756696	-0.648275
C	1.985218	0.202235	-2.237959
H	1.989393	1.076736	-1.584880
H	2.866988	0.225693	-2.881040
H	1.087718	0.260532	-2.857839
C	5.537569	-1.005307	-0.482976
H	5.608594	-1.885780	0.161118
C	6.692596	-0.949325	-1.470149
H	6.679793	-1.839378	-2.102885
H	6.610398	-0.062948	-2.104334
H	7.640662	-0.916107	-0.928553
C	6.425115	1.178332	2.373676
H	6.357896	2.129472	1.840553
H	7.412819	1.083530	2.827151
O	4.333071	-1.066020	-1.243806
O	6.371057	0.101993	1.413559
C	3.204458	-1.191678	-0.526417
C	5.506094	0.236111	0.407690
O	3.188121	-1.357086	0.668391
O	4.821753	1.210620	0.202842
C	5.318871	1.035866	3.401873
H	4.338006	1.106248	2.926280
H	5.390068	0.074779	3.917213
H	5.402255	1.836039	4.143400

Complex P2

C	2.128072	-2.413326	-0.892477
O	0.118461	-1.585051	0.188060
C	0.556066	-1.332625	-2.545904
C	1.162600	-2.508828	0.288289
O	3.240590	-2.877716	-0.821394

H	0.825933	-0.958173	-3.543392
C	-0.038073	-0.134123	-1.808069
O	-1.240864	0.000392	-1.673748
C	0.711678	-3.958690	0.474078
H	1.586452	-4.602624	0.587748
H	0.088485	-4.026467	1.369419
H	0.125341	-4.303835	-0.383020
H	1.793648	-2.230142	1.141711
C	-4.265317	-0.298455	-0.670230
C	-4.269248	0.970914	-1.181993
N	-4.653829	-0.543743	0.593457
C	-4.624158	2.068161	-0.351694
H	-3.896020	1.115578	-2.185119
C	-5.052551	0.459987	1.395230
H	-4.564001	-1.524578	0.967386
C	-5.068187	1.757134	0.963418
N	-4.509661	3.342664	-0.774344
H	-5.331516	0.180360	2.403455
H	-5.347102	2.531024	1.663278
C	-3.898326	3.625824	-2.066640
C	-4.735409	4.441629	0.158129
H	-4.462551	3.153300	-2.877166
H	-3.912158	4.701718	-2.233220
H	-2.855859	3.288281	-2.086827
H	-4.041414	4.382508	1.003909
H	-4.568227	5.382955	-0.362802
H	-5.768663	4.437116	0.521437
O	-4.326176	-3.032496	1.461964
S	-2.942566	-3.532894	1.190876
O	-2.081570	-3.617387	2.373910
O	-2.327715	-2.774468	0.054572
C	-3.169221	-5.200263	0.587661
H	-3.653942	-5.780526	1.373180
H	-3.791242	-5.168483	-0.307076
H	-2.185369	-5.613590	0.361162
H	-0.758284	-2.022295	0.263533
O	-1.838684	1.155377	0.681157
H	-3.899375	-1.155978	-1.221637
H	-1.579933	0.810786	-0.215259
O	0.743676	0.867280	-1.406523

C	2.105317	1.061994	-1.802038
H	2.309602	0.573606	-2.756643
S	-1.254288	2.606394	0.955269
O	-2.138477	3.216125	1.937609
O	1.829981	-1.740265	-2.039397
C	-0.436082	-2.466738	-2.732430
H	-1.242453	-2.134786	-3.390132
H	0.078140	-3.305433	-3.208653
H	-0.888269	-2.782523	-1.792317
C	0.306341	2.245390	1.721102
H	0.894943	1.610058	1.056426
H	0.805645	3.197006	1.908873
H	0.099477	1.725358	2.656371
O	-1.037627	3.291983	-0.313805
C	2.315880	2.566488	-1.907550
H	2.059351	3.046739	-0.960966
H	3.361731	2.777643	-2.141740
H	1.672814	2.975681	-2.689077
C	5.209068	-0.320728	-0.395770
H	4.759087	-1.060966	0.270539
C	6.294373	-0.962406	-1.243368
H	5.854755	-1.784478	-1.811325
H	6.729992	-0.234598	-1.933235
H	7.079502	-1.358363	-0.596109
C	7.033394	1.368842	2.345958
H	7.349416	2.243310	1.772408
H	7.908459	0.878036	2.774607
O	4.209085	0.170961	-1.290416
O	6.488887	0.389515	1.437518
C	3.033176	0.478814	-0.748662
C	5.726091	0.843935	0.441409
O	2.749305	0.345713	0.418622
O	5.493587	2.009392	0.220764
C	6.020985	1.740262	3.412794
H	5.149475	2.224292	2.966252
H	5.692914	0.853056	3.960538
H	6.476820	2.436979	4.122784

For DMAP:MSA (1:2) – Initiation

Complex I1

C	3.801527	1.730157	0.326266
O	2.119502	0.285783	1.308150
C	3.805213	-0.610356	-0.158495
C	2.310317	1.500576	0.538481
O	4.333419	2.807299	0.364294
H	3.242009	-0.527147	-1.096611
C	2.759686	-0.812096	0.920258
O	2.490996	-1.880731	1.432891
C	1.619443	2.619744	1.278227
H	1.771283	3.553592	0.734832
H	0.550038	2.404099	1.314188
H	2.030970	2.733475	2.284196
H	1.826669	1.352268	-0.434937
C	-1.005841	-2.242422	0.686811
C	-2.307842	-1.942808	0.418784
N	-0.312308	-1.575107	1.632588
C	-2.960381	-0.892176	1.129322
H	-2.804026	-2.472222	-0.380600
C	-0.881404	-0.560695	2.314954
H	0.692442	-1.760008	1.734255
C	-2.183954	-0.210904	2.112075
N	-4.232870	-0.559502	0.878029
H	-0.235813	-0.031035	3.003376
H	-2.576735	0.643599	2.642311
C	-4.908964	-1.112539	-0.291689
C	-4.845387	0.568248	1.571495
H	-5.033256	-2.196528	-0.194047
H	-5.899314	-0.666478	-0.365579
H	-4.341784	-0.869961	-1.196891
H	-4.780657	0.431047	2.655312
H	-4.358957	1.502243	1.271241
H	-5.900767	0.609425	1.305749
O	-2.756850	2.053512	-0.211893
S	-1.867956	1.426056	-1.208987
O	-2.433165	0.230801	-1.873798
O	-0.498095	1.161419	-0.659166
C	-1.621355	2.647793	-2.495026

H	-2.593777	2.883893	-2.928297
H	-1.177794	3.536362	-2.044751
H	-0.958715	2.225659	-3.251410
H	0.317680	-0.248323	-1.081199
H	-0.456521	-2.999911	0.144150
O	0.940573	-0.993270	-1.250208
C	0.527144	-1.691903	-2.407663
H	0.538494	-1.027995	-3.283631
H	-0.508978	-2.041143	-2.290587
C	1.468381	-2.862830	-2.624505
H	1.170767	-3.444757	-3.502029
H	2.493566	-2.510411	-2.779798
H	1.468411	-3.524872	-1.751925
O	4.519394	0.616350	0.080218
C	4.811939	-1.734596	-0.234364
H	4.293482	-2.671409	-0.446061
H	5.525448	-1.528759	-1.034471
H	5.352080	-1.837821	0.709190

TS I1

C	-3.857765	-0.117396	-1.200025
O	-1.496996	-0.820243	-0.748494
C	-3.311580	-1.608045	0.622621
C	-2.378570	-0.029175	-1.562476
O	-4.682930	0.464590	-1.858585
H	-3.660907	-1.560876	1.656386
C	-1.888320	-1.058850	0.558418
O	-1.036834	-1.683524	1.270506
C	-2.153451	-0.444193	-3.007719
H	-2.776107	0.166379	-3.662988
H	-1.104631	-0.290289	-3.272672
H	-2.403418	-1.499208	-3.154568
H	-2.109731	1.027465	-1.434273
C	2.139471	-0.397577	1.544465
C	3.457233	-0.138670	1.261668
N	1.439486	-1.320973	0.866206
C	4.109129	-0.846523	0.219600
H	3.956883	0.637543	1.823345

C	2.024715	-2.018311	-0.118136
H	0.353230	-1.448039	1.045118
C	3.339333	-1.826013	-0.464512
N	5.393891	-0.588416	-0.116859
H	1.388751	-2.729129	-0.633198
H	3.752271	-2.410438	-1.274803
C	6.119439	0.484714	0.549154
C	6.025431	-1.317283	-1.205328
H	6.233243	0.281812	1.619746
H	7.114131	0.564969	0.113699
H	5.611660	1.446991	0.420910
H	6.026203	-2.395448	-1.011572
H	5.522969	-1.128332	-2.161536
H	7.061074	-0.994051	-1.297129
O	1.539253	3.311523	-0.051964
S	0.489680	2.349437	-0.380841
O	-0.730985	2.841755	-1.027923
O	0.137786	1.503075	0.849656
C	1.214868	1.162954	-1.512085
H	1.371021	1.670975	-2.464673
H	2.168395	0.828779	-1.102453
H	0.525459	0.324809	-1.621866
H	-1.054074	0.983234	0.906987
H	1.594773	0.157208	2.295609
O	-2.040435	0.505922	1.089139
C	-2.397687	0.699343	2.467536
H	-1.666296	1.397600	2.885008
H	-2.279248	-0.257726	2.987875
C	-3.803782	1.256819	2.574738
H	-4.062708	1.408536	3.627339
H	-3.870915	2.216781	2.057092
H	-4.537996	0.581200	2.128483
O	-4.245214	-0.816149	-0.121837
C	-3.352299	-3.046398	0.129291
H	-2.722980	-3.671973	0.764988
H	-4.380627	-3.413229	0.159364
H	-2.985097	-3.108773	-0.899432

Int. 1a

C	3.721762	1.024167	-0.970199
O	1.571857	0.570567	0.188292
C	3.618336	-0.250475	1.087371
C	2.202901	1.206827	-0.927631
O	4.358226	1.486221	-1.884717
H	4.172145	-1.166251	1.311028
C	2.210931	-0.631230	0.612106
O	1.533184	-1.172182	1.646488
C	1.855625	2.687535	-0.893110
H	2.372498	3.198688	-1.707023
H	0.779773	2.808685	-1.032107
H	2.164657	3.131127	0.058763
H	1.826110	0.755134	-1.850775
C	-1.844733	-2.119272	1.481412
C	-3.129063	-1.824374	1.072317
N	-0.770021	-2.076333	0.682091
C	-3.360103	-1.428690	-0.264282
H	-3.925999	-1.847913	1.803156
C	-0.991615	-1.745550	-0.598466
H	0.681061	-1.599977	1.303308
C	-2.231930	-1.430546	-1.114420
N	-4.594516	-1.046546	-0.704341
H	-0.106760	-1.688700	-1.227174
H	-2.299475	-1.128391	-2.150405
C	-5.643935	-0.808074	0.271665
C	-4.701948	-0.382463	-1.993169
H	-5.367045	-0.012875	0.976205
H	-6.553344	-0.511625	-0.251306
H	-5.870987	-1.722078	0.829275
H	-4.389039	-1.049625	-2.802458
H	-5.745103	-0.120460	-2.170403
H	-4.097690	0.533355	-2.029065
O	-3.244114	1.599468	0.548480
S	-1.877040	1.967452	0.232801
O	-1.415688	1.922416	-1.148180
O	-0.924077	1.077544	1.141801
C	-1.579170	3.599705	0.882264
H	-2.217736	4.289810	0.329615
H	-1.837662	3.597955	1.940917
H	-0.527689	3.848562	0.736075

H	-0.047600	0.887727	0.713413
H	-1.655377	-2.376977	2.520066
O	2.225241	-1.434261	-0.538737
C	2.778110	-2.748518	-0.416469
H	2.024379	-3.433996	-0.816390
H	2.909939	-2.998450	0.641404
C	4.078598	-2.858990	-1.192804
H	4.462576	-3.882499	-1.135552
H	3.917184	-2.608702	-2.244763
H	4.838114	-2.178134	-0.798941
O	4.349028	0.358093	0.008210
C	3.616507	0.664554	2.297476
H	3.147170	0.157706	3.142551
H	4.643856	0.929359	2.557598
H	3.055564	1.578891	2.086450

TS I2

C	3.574265	1.011145	-0.958314
O	1.656099	0.673516	0.492309
C	3.714968	-0.388117	1.057779
C	2.111591	1.367935	-0.703988
O	4.120309	1.405564	-1.959512
H	4.318682	-1.300316	1.131524
C	2.295980	-0.878841	0.790964
O	1.665130	-1.426302	1.715647
C	1.958670	2.879292	-0.587281
H	2.432284	3.354225	-1.448397
H	0.898118	3.132585	-0.596422
H	2.428085	3.244718	0.330783
H	1.547409	1.002769	-1.567000
C	-1.848288	-1.942936	1.612627
C	-3.124293	-1.728585	1.172794
N	-0.798530	-1.911200	0.772761
C	-3.356996	-1.401036	-0.191579
H	-3.922630	-1.724665	1.900031
C	-0.983879	-1.654209	-0.535935
H	0.177291	-1.861295	1.162927
C	-2.227754	-1.432310	-1.053451

N	-4.577444	-1.047598	-0.641147
H	-0.078891	-1.576654	-1.126607
H	-2.304916	-1.174199	-2.098532
C	-5.630011	-0.756226	0.323168
C	-4.691628	-0.435927	-1.960934
H	-5.344876	0.087274	0.963563
H	-6.540899	-0.500121	-0.216457
H	-5.848828	-1.634331	0.937271
H	-4.376326	-1.134757	-2.740930
H	-5.736187	-0.189142	-2.144281
H	-4.092000	0.480507	-2.011318
O	-3.189713	1.415977	0.275401
S	-1.801929	1.892253	0.150602
O	-1.297028	2.021650	-1.227005
O	-0.849949	1.082640	1.007332
C	-1.782583	3.526126	0.881724
H	-2.438598	4.165336	0.289817
H	-2.147416	3.444386	1.905956
H	-0.762142	3.909643	0.870275
H	0.561855	0.849650	0.715008
H	-1.617051	-2.113315	2.656539
O	2.120628	-1.371502	-0.517596
C	2.644138	-2.687527	-0.747007
H	1.839896	-3.261995	-1.220661
H	2.862442	-3.168405	0.212996
C	3.864580	-2.637394	-1.649347
H	4.207852	-3.653864	-1.868095
H	3.621101	-2.142118	-2.593025
H	4.683707	-2.083272	-1.184140
O	4.285962	0.306973	-0.062979
C	3.863959	0.419893	2.331811
H	3.500347	-0.167815	3.177552
H	4.915849	0.667868	2.488285
H	3.279549	1.340145	2.271877

Complex I2

C	2.696005	2.168279	0.023730
O	1.300669	0.283931	-0.731723

C	2.965873	0.200733	1.580990
C	2.031929	1.413953	-1.140149
O	2.872892	3.359785	-0.014362
H	3.818467	0.031822	2.253277
C	3.077022	-0.943785	0.583994
O	2.511512	-2.017061	0.731245
C	1.205823	2.351604	-2.009127
H	1.811311	3.184997	-2.368088
H	0.810697	1.788971	-2.858078
H	0.360280	2.751551	-1.446362
H	2.878955	1.034886	-1.726264
C	-1.182455	-2.068433	1.453896
C	-2.509950	-1.894796	1.199007
N	-0.271702	-2.093846	0.458646
C	-2.957034	-1.684119	-0.138614
H	-3.187781	-1.821323	2.035832
C	-0.653712	-1.927253	-0.825193
H	0.731226	-2.125323	0.674094
C	-1.962007	-1.739589	-1.157385
N	-4.240578	-1.428825	-0.424950
H	0.143647	-1.900462	-1.555084
H	-2.205439	-1.548604	-2.191510
C	-5.204669	-1.249132	0.654751
C	-4.603458	-0.972585	-1.764694
H	-4.945154	-0.363045	1.244108
H	-6.194705	-1.117107	0.220675
H	-5.237727	-2.138296	1.291833
H	-4.409169	-1.754245	-2.506632
H	-5.669931	-0.754184	-1.781263
H	-4.047112	-0.061048	-2.012149
O	-3.266188	1.182908	1.054470
S	-2.185348	1.643656	0.160024
O	-2.452493	1.435788	-1.279087
O	-0.846819	1.099831	0.569482
C	-2.059336	3.411749	0.412518
H	-3.016612	3.858288	0.141707
H	-1.835810	3.590488	1.464495
H	-1.262734	3.806069	-0.219121
H	0.438395	0.591413	-0.351520
H	-0.794086	-2.163446	2.459716

O	3.977386	-0.712876	-0.357236
C	4.179046	-1.735622	-1.353290
H	4.547838	-1.188263	-2.222393
H	3.211779	-2.183822	-1.591585
C	5.179741	-2.773481	-0.883831
H	5.366261	-3.489949	-1.689401
H	6.128930	-2.301062	-0.617665
H	4.796233	-3.319529	-0.019215
O	3.246080	1.499899	1.071245
C	1.695833	0.172675	2.414994
H	1.607421	-0.797400	2.911757
H	1.768082	0.946445	3.182117
H	0.802036	0.363535	1.818603

For DMAP:MSA (1:2) – Propagation

Complex P1

C	1.528858	-2.778181	0.724822
O	-0.281577	-1.513025	1.746999
C	-0.377390	-2.189011	-0.555999
C	1.156260	-1.543683	1.528366
O	2.547393	-3.402469	0.880425
H	0.134816	-1.340013	-1.021204
C	-1.058416	-1.667647	0.695139
O	-2.252900	-1.425450	0.789287
C	1.824609	-1.484989	2.880349
H	2.904941	-1.456107	2.735312
H	1.514425	-0.575407	3.398475
H	1.570058	-2.357015	3.487458
H	1.408270	-0.663219	0.927914
C	-3.834298	2.792104	-0.811400
C	-5.075325	2.494686	-0.320783
N	-2.714464	2.392421	-0.181185
C	-5.201066	1.745938	0.883983
H	-5.942106	2.807665	-0.884268
C	-2.786187	1.672398	0.954274
H	-1.769955	2.632897	-0.571304
C	-3.991477	1.346811	1.514853
N	-6.405132	1.420569	1.389354
H	-1.834691	1.343270	1.354724
H	-3.990145	0.723761	2.397328
C	-7.616924	1.709081	0.632927
C	-6.495061	0.623544	2.606023
H	-7.579498	1.229831	-0.350830
H	-8.472880	1.311648	1.175778
H	-7.760910	2.789264	0.516642
H	-6.089323	-0.380121	2.443142
H	-5.964133	1.113149	3.428900
H	-7.542285	0.533767	2.890598
O	-0.256019	3.060974	-1.054125
S	0.735798	2.020500	-0.649545
O	1.596607	1.568926	-1.766076
O	0.069367	0.905207	0.073287
C	1.818862	2.801156	0.533780

H	2.284057	3.662168	0.052974
H	1.221820	3.117581	1.389758
H	2.570355	2.065383	0.825312
H	2.084313	-0.107241	-1.660176
O	-3.934567	-0.739358	-1.059264
H	-3.690896	3.349731	-1.728518
H	-3.319424	-1.057915	-0.327782
O	2.263110	-1.068769	-1.569875
C	3.640629	-1.296240	-1.546721
H	3.788506	-2.380741	-1.571487
S	-5.400004	-1.325898	-0.920518
O	-6.284645	-0.408456	-1.618486
O	0.612072	-3.161518	-0.179232
C	-1.313108	-2.854579	-1.539928
H	-2.021025	-2.125264	-1.937825
H	-0.721367	-3.251213	-2.366658
H	-1.860592	-3.675178	-1.067889
C	-5.297285	-2.838474	-1.854109
H	-4.562716	-3.488177	-1.377478
H	-6.285505	-3.299376	-1.836735
H	-5.003376	-2.592673	-2.874386
O	-5.665413	-1.657419	0.472423
C	6.231257	-0.723903	1.018340
H	5.649923	-0.803123	1.940476
C	4.358707	-0.642334	-2.732350
H	4.208986	0.441694	-2.709717
H	5.430602	-0.851391	-2.708647
H	3.932349	-1.031268	-3.659754
C	7.531055	-1.505070	1.125455
H	7.311112	-2.557116	1.318707
H	8.102849	-1.429013	0.196965
H	8.129583	-1.108900	1.948848
C	6.530171	2.513506	-0.794733
H	7.305218	2.961113	-0.168115
H	6.849738	2.527305	-1.837929
C	5.190518	3.199982	-0.610051
H	4.901586	3.194604	0.443683
H	5.260689	4.239727	-0.944183
H	4.411431	2.698312	-1.191290
C	4.254238	-0.790722	-0.247680

C	6.499124	0.764129	0.805905
O	5.488353	-1.282053	-0.065239
O	6.423278	1.112013	-0.478356
O	6.790706	1.506468	1.715167
O	3.733380	-0.008567	0.518594

TS P1

C	-0.053885	-4.249565	-0.664764
O	-1.226914	-2.195076	0.057653
C	-0.763396	-2.543198	-2.245890
C	-0.597928	-3.403312	0.487430
O	0.459725	-5.318182	-0.448991
H	-0.216780	-2.160605	-3.110971
C	-0.605929	-1.571346	-1.069821
O	-1.263084	-0.425573	-1.339897
C	-1.601319	-4.192688	1.312218
H	-1.128652	-5.101998	1.685669
H	-1.936863	-3.584081	2.155176
H	-2.464290	-4.475915	0.699367
H	0.272977	-3.138759	1.096053
C	-3.682870	1.547232	-1.017382
C	-2.931729	2.461380	-1.696894
N	-4.136065	1.799827	0.226598
C	-2.565352	3.687715	-1.071757
H	-2.542023	2.181088	-2.664067
C	-3.839161	2.962193	0.846354
H	-4.625691	1.065259	0.745034
C	-3.091097	3.920214	0.232876
N	-1.746087	4.569147	-1.663166
H	-4.202023	3.069227	1.860107
H	-2.829038	4.803655	0.794451
C	-1.097979	4.220619	-2.921506
C	-1.202123	5.682015	-0.887548
H	-1.843707	3.987942	-3.687810
H	-0.523024	5.078008	-3.268173
H	-0.418962	3.373931	-2.770465
H	-0.678170	5.305225	-0.001788
H	-0.496399	6.228075	-1.511097

H	-1.998678	6.373655	-0.593351
O	-5.272965	-0.342798	1.712462
S	-4.392754	-1.512228	1.732170
O	-3.380175	-1.631397	2.761622
O	-3.739719	-1.589760	0.290279
C	-5.428786	-2.956508	1.749613
H	-5.980973	-2.943991	2.689950
H	-6.110037	-2.906765	0.900698
H	-4.787597	-3.836350	1.692605
H	-2.750899	-1.789630	0.300093
O	-1.030810	1.234462	0.622613
H	-3.907284	0.566637	-1.416142
H	-1.109236	0.244017	-0.593941
O	0.717412	-1.398694	-0.655314
C	1.759414	-1.251091	-1.603549
H	1.803844	-2.135009	-2.256725
S	0.058739	2.270951	0.609588
O	-0.294487	3.463479	1.403454
O	-0.117348	-3.788967	-1.927461
C	-2.209983	-2.789868	-2.632643
H	-2.672811	-1.853611	-2.950147
H	-2.247864	-3.508437	-3.454403
H	-2.778756	-3.189500	-1.788982
C	1.446641	1.498557	1.429626
H	1.639287	0.531753	0.963236
H	2.309180	2.157553	1.317921
H	1.181948	1.362766	2.479400
O	0.499443	2.574446	-0.771322
C	1.664218	0.024716	-2.436562
H	1.596597	0.907234	-1.795146
H	2.541148	0.103957	-3.082138
H	0.765461	-0.000712	-3.057352
C	5.361672	-1.073777	-0.847938
H	5.496485	-1.974780	-0.244136
C	6.457468	-0.933421	-1.890993
H	6.446289	-1.797687	-2.558681
H	6.306361	-0.025690	-2.480416
H	7.431867	-0.882690	-1.398854
C	6.098825	0.949776	2.159961
H	6.066599	1.937337	1.695493

H	7.030470	0.827383	2.713134
C	4.894982	0.744371	3.071027
H	4.930396	-0.260685	3.507572
H	3.976558	0.825968	2.481246
O	4.118347	-1.153013	-1.547497
O	6.114632	-0.071330	1.147203
O	4.958338	1.722708	4.095013
H	4.106454	2.154824	4.179884
C	3.032710	-1.291679	-0.769346
C	5.327883	0.133625	0.087270
O	3.086658	-1.434388	0.427700
O	4.700131	1.141992	-0.127909

Int. P1

C	-0.591438	-3.804783	0.298468
O	-1.335885	-1.641013	1.289603
C	-1.267384	-1.930283	-1.069572
C	-0.892926	-2.966678	1.543544
O	-0.198003	-4.940366	0.412209
H	-0.855729	-1.551621	-2.006371
C	-0.824880	-1.070610	0.122800
O	-1.292098	0.192805	0.112810
C	-1.948345	-3.642132	2.406387
H	-1.618108	-4.644539	2.683063
H	-2.105199	-3.050562	3.311762
H	-2.896507	-3.719396	1.865604
H	0.058349	-2.926598	2.088448
C	-2.549132	1.325896	2.730276
C	-3.582539	0.535123	2.321472
N	-2.143668	2.378305	1.993017
C	-4.209776	0.781145	1.067758
H	-3.851934	-0.310885	2.936296
C	-2.732060	2.667304	0.811876
H	-1.296617	2.861339	2.297264
C	-3.773715	1.922932	0.340005
N	-5.153167	-0.044876	0.581805
H	-2.304265	3.481670	0.242639
H	-4.151458	2.143701	-0.649879

C	-5.540052	-1.227385	1.333329
C	-5.819377	0.251405	-0.686137
H	-6.037398	-0.965562	2.275429
H	-6.231624	-1.817895	0.734701
H	-4.666018	-1.849867	1.554329
H	-6.450586	1.143241	-0.594238
H	-5.094474	0.410060	-1.490485
H	-6.453401	-0.592918	-0.952796
O	0.439273	2.747173	3.163286
S	1.305535	1.906115	2.331485
O	1.439634	2.253738	0.926813
O	0.762796	0.433750	2.500930
C	2.911220	1.824808	3.081270
H	3.352221	2.820057	3.015070
H	2.788286	1.520461	4.120357
H	3.492685	1.093409	2.518426
H	0.864131	-0.105356	1.663429
O	-1.441141	1.754154	-1.958053
H	-1.987880	1.140315	3.636617
H	-1.254811	0.676272	-0.760521
O	0.606212	-1.098864	0.321045
C	1.512958	-1.088381	-0.778741
H	1.386641	-2.007320	-1.361277
S	-2.238149	1.251239	-3.127155
O	-3.694949	1.301982	-2.866589
O	-0.719902	-3.258522	-0.917254
C	-2.777575	-1.999130	-1.200669
H	-3.180097	-1.005313	-1.401186
H	-3.030660	-2.641741	-2.045632
H	-3.230929	-2.395925	-0.287779
C	-1.922889	2.428042	-4.435038
H	-0.852107	2.433270	-4.639971
H	-2.481171	2.107533	-5.315080
H	-2.257937	3.411509	-4.104678
O	-1.742306	-0.056643	-3.598147
C	5.157107	-1.576243	-0.563959
H	5.255919	-1.965873	0.452259
C	1.418988	0.144777	-1.678219
H	1.451809	1.052877	-1.073630
H	2.262176	0.138471	-2.373888

H	0.497420	0.144261	-2.262365
C	5.963330	-2.407602	-1.549135
H	5.618183	-3.443313	-1.526193
H	5.847521	-2.019885	-2.564448
H	7.019117	-2.377698	-1.271708
C	5.265287	2.086125	-1.248896
H	6.350790	2.166041	-1.344351
H	4.788495	2.477968	-2.148316
C	4.755550	2.781132	-0.001623
H	5.264285	2.395111	0.885558
H	4.960684	3.853671	-0.074936
H	3.676869	2.639553	0.105979
C	2.888566	-1.119112	-0.139297
C	5.660299	-0.135943	-0.527871
O	3.786827	-1.653679	-0.970065
O	4.903018	0.688969	-1.245592
O	6.671200	0.173057	0.060555
O	3.158535	-0.678121	0.956110

Int. P2

C	0.212079	-4.177425	-0.698633
O	-1.071800	-2.190497	0.021903
C	-0.461313	-2.438210	-2.259864
C	-0.425489	-3.390557	0.447731
O	0.747209	-5.237013	-0.489830
H	0.121868	-2.007084	-3.076924
C	-0.407000	-1.505134	-1.043658
O	-1.086304	-0.372537	-1.312558
C	-1.445137	-4.240976	1.187474
H	-0.962493	-5.146232	1.558015
H	-1.849078	-3.673126	2.028891
H	-2.261617	-4.531766	0.517136
H	0.400011	-3.117965	1.113461
C	-3.591856	1.499182	-1.067731
C	-2.824024	2.451918	-1.671370
N	-4.125719	1.700730	0.153335
C	-2.526199	3.665108	-0.986939
H	-2.369334	2.211039	-2.620577

C	-3.897093	2.849566	0.825107
H	-4.622955	0.936734	0.619145
C	-3.138230	3.844244	0.288090
N	-1.691204	4.582041	-1.497027
H	-4.322263	2.915302	1.817921
H	-2.933007	4.714550	0.892050
C	-0.951463	4.284474	-2.717317
C	-1.222578	5.679548	-0.653078
H	-1.637707	4.058253	-3.539105
H	-0.374637	5.163827	-2.999968
H	-0.264264	3.448850	-2.543115
H	-0.750602	5.284723	0.253835
H	-0.488472	6.257882	-1.211330
H	-2.051075	6.346541	-0.391960
O	-5.271268	-0.530466	1.497660
S	-4.352062	-1.669254	1.530335
O	-3.400203	-1.793048	2.615442
O	-3.611517	-1.671894	0.129538
C	-5.335119	-3.147257	1.433609
H	-5.942924	-3.188113	2.338158
H	-5.965471	-3.089084	0.546752
H	-4.660613	-4.002355	1.385298
H	-2.618473	-1.839463	0.192148
O	-1.034899	1.205139	0.720874
H	-3.766715	0.526880	-1.509243
H	-1.002043	0.272589	-0.532562
O	0.882798	-1.306293	-0.544543
C	1.971477	-1.085943	-1.423555
H	2.081857	-1.939155	-2.108856
S	0.021233	2.271433	0.833648
O	-0.431066	3.418758	1.644068
O	0.206947	-3.673656	-1.946236
C	-1.873222	-2.717333	-2.741103
H	-2.348337	-1.785553	-3.053601
H	-1.838076	-3.406687	-3.587674
H	-2.476477	-3.165081	-1.947009
C	1.366897	1.503601	1.722683
H	1.630552	0.569522	1.225623
H	2.211000	2.194385	1.716689
H	1.028959	1.306776	2.740767

O	0.549791	2.648166	-0.498249
C	1.881286	0.220725	-2.207770
H	1.743001	1.071204	-1.535166
H	2.792832	0.357139	-2.793102
H	1.023749	0.191807	-2.884357
C	5.524781	-0.866804	-0.481748
H	5.625760	-1.764637	0.133557
C	6.671770	-0.742022	-1.471504
H	6.686016	-1.611234	-2.132746
H	6.556457	0.161568	-2.075470
H	7.621223	-0.694235	-0.932949
C	6.278057	1.219641	2.483712
H	6.232591	2.187601	1.979258
H	7.242508	1.112489	2.982629
O	4.318755	-0.943248	-1.240505
O	6.275321	0.175276	1.487616
C	3.197980	-1.124682	-0.521818
C	5.459888	0.346203	0.444968
O	3.192932	-1.305175	0.670617
O	4.795576	1.331809	0.233908
C	5.121486	1.040674	3.449011
H	4.165970	1.121266	2.925918
H	5.172292	0.064326	3.937566
H	5.161647	1.818432	4.217609

TS P2

C	0.156738	-4.031379	-0.739707
O	-1.078932	-2.077003	0.142512
C	-0.569378	-2.239443	-2.240654
C	-0.480411	-3.341618	0.466519
O	0.745837	-5.072155	-0.586625
H	-0.006637	-1.827859	-3.083983
C	-0.418770	-1.260472	-1.070940
O	-1.044344	-0.158584	-1.195277
C	-1.498700	-4.255096	1.132597
H	-0.994150	-5.173797	1.434545
H	-1.922918	-3.759593	2.008434
H	-2.306726	-4.509465	0.439862

H	0.343689	-3.123704	1.150306
C	-3.599067	1.395564	-0.902171
C	-2.922869	2.412479	-1.517005
N	-4.096580	1.537675	0.339678
C	-2.691895	3.632149	-0.824297
H	-2.488973	2.220458	-2.486941
C	-3.924452	2.685785	1.023191
H	-4.552886	0.720529	0.792786
C	-3.253154	3.742762	0.480045
N	-1.956653	4.623442	-1.359566
H	-4.326542	2.706725	2.028001
H	-3.097942	4.621160	1.088353
C	-1.285279	4.418232	-2.636560
C	-1.578505	5.770809	-0.541249
H	-2.010460	4.178213	-3.420661
H	-0.781606	5.341025	-2.920457
H	-0.537690	3.621856	-2.552323
H	-1.022210	5.445554	0.344686
H	-0.941479	6.426838	-1.132130
H	-2.463192	6.342061	-0.239755
O	-5.210762	-0.675061	1.437372
S	-4.341982	-1.877353	1.431535
O	-3.528235	-2.084093	2.627786
O	-3.517473	-1.907562	0.154548
C	-5.448989	-3.266001	1.270967
H	-6.104002	-3.269665	2.142538
H	-6.026747	-3.148207	0.354420
H	-4.849675	-4.176553	1.240332
H	-2.154335	-2.006180	0.183406
O	-0.891309	1.360879	0.721932
H	-3.721637	0.417317	-1.349121
H	-0.889898	0.704895	-0.138926
O	0.880779	-1.210979	-0.542325
C	1.981331	-1.092343	-1.427264
H	2.024329	-1.962818	-2.098066
S	0.236174	2.436109	0.726182
O	-0.197352	3.544165	1.573451
O	0.083538	-3.493004	-1.967538
C	-2.004799	-2.469178	-2.673278
H	-2.457012	-1.514662	-2.949037

H	-2.017713	-3.137947	-3.536840
H	-2.603884	-2.909879	-1.873286
C	1.593520	1.606842	1.523436
H	1.745059	0.640423	1.041741
H	2.479611	2.233614	1.415028
H	1.329470	1.468541	2.572067
O	0.629957	2.756696	-0.648275
C	1.985218	0.202235	-2.237959
H	1.989393	1.076736	-1.584880
H	2.866988	0.225693	-2.881040
H	1.087718	0.260532	-2.857839
C	5.537569	-1.005307	-0.482976
H	5.608594	-1.885780	0.161118
C	6.692596	-0.949325	-1.470149
H	6.679793	-1.839378	-2.102885
H	6.610398	-0.062948	-2.104334
H	7.640662	-0.916107	-0.928553
C	6.425115	1.178332	2.373676
H	6.357896	2.129472	1.840553
H	7.412819	1.083530	2.827151
O	4.333071	-1.066020	-1.243806
O	6.371057	0.101993	1.413559
C	3.204458	-1.191678	-0.526417
C	5.506094	0.236111	0.407690
O	3.188121	-1.357086	0.668391
O	4.821753	1.210620	0.202842
C	5.318871	1.035866	3.401873
H	4.338006	1.106248	2.926280
H	5.390068	0.074779	3.917213
H	5.402255	1.836039	4.143400

Complex P2

C	2.128072	-2.413326	-0.892477
O	0.118461	-1.585051	0.188060
C	0.556066	-1.332625	-2.545904
C	1.162600	-2.508828	0.288289
O	3.240590	-2.877716	-0.821394
H	0.825933	-0.958173	-3.543392

C	-0.038073	-0.134123	-1.808069
O	-1.240864	0.000392	-1.673748
C	0.711678	-3.958690	0.474078
H	1.586452	-4.602624	0.587748
H	0.088485	-4.026467	1.369419
H	0.125341	-4.303835	-0.383020
H	1.793648	-2.230142	1.141711
C	-4.265317	-0.298455	-0.670230
C	-4.269248	0.970914	-1.181993
N	-4.653829	-0.543743	0.593457
C	-4.624158	2.068161	-0.351694
H	-3.896020	1.115578	-2.185119
C	-5.052551	0.459987	1.395230
H	-4.564001	-1.524578	0.967386
C	-5.068187	1.757134	0.963418
N	-4.509661	3.342664	-0.774344
H	-5.331516	0.180360	2.403455
H	-5.347102	2.531024	1.663278
C	-3.898326	3.625824	-2.066640
C	-4.735409	4.441629	0.158129
H	-4.462551	3.153300	-2.877166
H	-3.912158	4.701718	-2.233220
H	-2.855859	3.288281	-2.086827
H	-4.041414	4.382508	1.003909
H	-4.568227	5.382955	-0.362802
H	-5.768663	4.437116	0.521437
O	-4.326176	-3.032496	1.461964
S	-2.942566	-3.532894	1.190876
O	-2.081570	-3.617387	2.373910
O	-2.327715	-2.774468	0.054572
C	-3.169221	-5.200263	0.587661
H	-3.653942	-5.780526	1.373180
H	-3.791242	-5.168483	-0.307076
H	-2.185369	-5.613590	0.361162
H	-0.758284	-2.022295	0.263533
O	-1.838684	1.155377	0.681157
H	-3.899375	-1.155978	-1.221637
H	-1.579933	0.810786	-0.215259
O	0.743676	0.867280	-1.406523
C	2.105317	1.061994	-1.802038

H	2.309602	0.573606	-2.756643
S	-1.254288	2.606394	0.955269
O	-2.138477	3.216125	1.937609
O	1.829981	-1.740265	-2.039397
C	-0.436082	-2.466738	-2.732430
H	-1.242453	-2.134786	-3.390132
H	0.078140	-3.305433	-3.208653
H	-0.888269	-2.782523	-1.792317
C	0.306341	2.245390	1.721102
H	0.894943	1.610058	1.056426
H	0.805645	3.197006	1.908873
H	0.099477	1.725358	2.656371
O	-1.037627	3.291983	-0.313805
C	2.315880	2.566488	-1.907550
H	2.059351	3.046739	-0.960966
H	3.361731	2.777643	-2.141740
H	1.672814	2.975681	-2.689077
C	5.209068	-0.320728	-0.395770
H	4.759087	-1.060966	0.270539
C	6.294373	-0.962406	-1.243368
H	5.854755	-1.784478	-1.811325
H	6.729992	-0.234598	-1.933235
H	7.079502	-1.358363	-0.596109
C	7.033394	1.368842	2.345958
H	7.349416	2.243310	1.772408
H	7.908459	0.878036	2.774607
O	4.209085	0.170961	-1.290416
O	6.488887	0.389515	1.437518
C	3.033176	0.478814	-0.748662
C	5.726091	0.843935	0.441409
O	2.749305	0.345713	0.418622
O	5.493587	2.009392	0.220764
C	6.020985	1.740262	3.412794
H	5.149475	2.224292	2.966252
H	5.692914	0.853056	3.960538
H	6.476820	2.436979	4.122784
